# Metal–Organic Frameworks for Electrocatalysis: Beyond Their Derivatives

**DOI:** 10.1002/smsc.202100015

**Published:** 2021-08-03

**Authors:** Yongchao Yang, Yuwei Yang, Yangyang Liu, Shenlong Zhao, Zhiyong Tang

**Affiliations:** ^1^ School of Chemical and Biomolecular Engineering The University of Sydney Camperdown NSW 2006 Australia; ^2^ CAS Key Laboratory of Nanosystem and Hierarchical Fabrication CAS Center for Excellence in Nanoscience National Center for Nanoscience and Technology Beijing 100190 P. R. China

**Keywords:** CO_2_ reduction, electrocatalysis, host–guest composites, hydrogen oxidation, metal–organic frameworks, oxygen reduction, water splitting

## Abstract

Electrocatalysis is at the heart of many significant chemical transformation processes and advanced clean energy technologies. Traditional noble/transition metal oxides are widely used as electrocatalysts; however, they often suffer from intrinsic disadvantages, including low atom utilization, small surface area, and unfavorable tunability. Metal–organic frameworks (MOFs), as a new family of catalytic materials, are attracting extensive attention due to their unique physicochemical properties. The tremendous pristine MOF‐based materials are created using various synthetic approaches and further used for important energy conversions. Herein, a systematic overview on the unique merits and the state‐of‐the‐art design of MOF‐based electrocatalysts is offered. This review also presents recent advances in the development of various pristine MOFs and MOF‐based host–guest composite catalysts for electrocatalysis (i.e., oxygen reduction reaction, hydrogen oxidation reaction, hydrogen evolution reaction, oxygen evolution reaction, and CO_2_ reduction reaction) and discusses the future challenges and opportunities in this emerging field.

## Graphical abstract

Metal–organic frameworks (MOFs) are a new family of catalytic materials, holding great promise in many energy‐conversion processes due to their unique physicochemical properties. This review systematically outlines the distinctive superiorities and state‐of‐the‐art development of MOF‐based catalysts. A critical summary is provided on their recent advances in electrocatalysis. Future key challenges and opportunities are suggested.

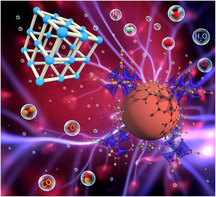

## 1 Introduction

Electrocatalysts play a key role in many energy conversion technologies, such as fuel cells, electrolyzers, and artificial CO_2_/N_2_ fixation.^[^ ^1^ ^]^ In the past decades, various nanomaterials have been fabricated to accelerate the related electrocatalytic processes, thereby promoting their energy conversion efficiency.^[^ ^2^ ^]^ Among them, noble metals, such as platinum (Pt), palladium (Pd), ruthenium (Ru), etc., are known to have high activities in electrocatalysis, but their scarcity and high price both greatly limit their corresponding practical application.^[^ ^3^ ^]^ As alternatives, a variety of metal oxides/sulfides/phosphides are attempted to reduce the cost and simultaneously improve the activity and durability.^[^ ^4^ ^]^ Substantial advances have been achieved so far; nevertheless, the activities of the majority of earth‐abundant metal‐based materials still cannot compete with the noble metal‐based catalysts. Therefore, it is highly desirable to develop readily available and cost‐effective electrocatalytic materials that possess the catalytic performances comparable to, or even better than, noble‐metal‐based ones.^[^ ^5^ ^]^

Beyond conventional electrocatalytic materials, metal–organic frameworks (MOFs) constructed by coordination of transition metal nodes with organic linkers have aroused intensive attention in the field of energy conversion and storage because of their intriguing features, including, but not limited to well‐defined structures, precisely controllable components and high surface area.^[^ ^6^ ^]^ Such distinctive features endow MOF‐based materials with the advantages of both heterogeneous and homogenous catalysts. For instance, periodic atom arrangement not only gives rise to the maximum utilization of metal atoms but also facilitates the identification of activity origin. In addition, the interaction between metal active centers and adjacent coordinating atoms is easily regulated, which is vital for the optimization of catalytic activity, selectivity, and durability of MOFs in various key reactions involved in the energy conversion processes. Last but not least, the MOFs with regular channels or pores allow efficient mass transfer and adequate access between active sites and reactants, which drastically reduce the cost of electric energy caused by interfacial resistance.

**Figure 1:**
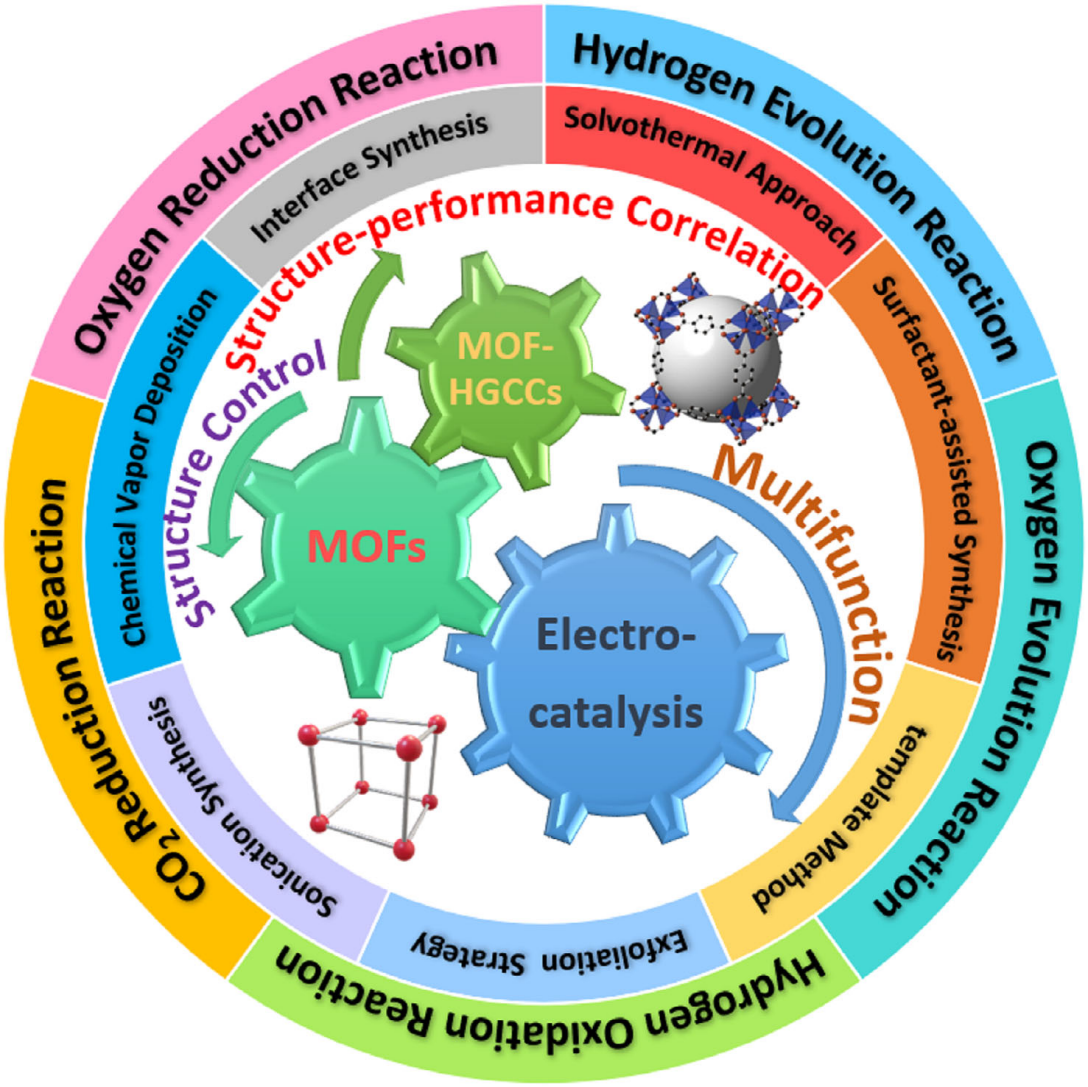
Overview of the development of pristine MOFs and MOF‐HGGCs for electrocatalysis involved in various key reactions. It can be expected that through the rational structure control in catalyst design and further structure–performance relationship exploration at the atomic or molecular level, ones can achieve the future development of high‐efficiency multifunctional pristine MOF‐based materials with exceptional catalytic activities, excellent selectivity, and ultralong‐term stability for large‐scale energy‐related practical applications.

Benefiting from the intensive research efforts on the development of novel electrocatalysts, many approaches, including size tailoring, defect engineering, and species hybrid, have been developed for designing high‐performance MOF‐based electrocatalysts. In 2016, Zhao et al., for the first time, reported a series of ultrathin MOF nanosheets (UMOFNs) for the electrocatalytic oxygen evolution reaction (OER) with excellent activity and stability.^[^ ^7^ ^]^ Since then, MOFs have been broadly examined as electrocatalysts, and some of them were able to exhibit better performances than commercial noble metal‐based electrocatalysts in oxygen reduction reaction (ORR), hydrogen oxidation reaction (HOR), hydrogen evolution reaction (HER), OER, or CO_2_ reduction reaction (CO_2_RR).^[^ ^8^ ^]^ Furthermore, MOFs have displayed the effective bifunctional catalysis for ORR/OER in metal–air batteries, HER/OER in overall electrolyzers, and other important electrochemical processes in related devices.^[^ ^9^ ^]^ These achievements make it possible for MOF‐based electrocatalysts to overtake the traditional metal‐based ones in the race to the renewable energy marketplace. Most importantly, the fundamental issues about the activity origin of MOF‐based electrocatalysts are clearly unveiled using operando X‐ray absorption spectroscopy (XAS), providing the atomic‐level understanding of the structure–performance correlation.^[^ ^10^ ^]^ Therefore, a timely review on this rapidly growing field is highly desired. Rather than a brief summary, this article aims to offer a comprehensive and critical overview of recent progress by summarizing the numerous representative works on pristine MOF‐based electrocatalysis and highlight the associated reaction mechanisms and design strategies. Through this systematic review, we hope to inspire the researchers to deeply understand the background, current research status, and future challenges of MOF‐based electrocatalysts (**Figure** **1**).

## 2 The Advantages of Pristine MOF‐Based Catalysts

The emerging MOFs, formed by coordination bonds between organic ligands and metal atom nodes, are of many exciting features, including structural designability, framework adaptivity and/or flexibility, ordered crystalline pores, and homogenous and periodic distribution of constituent elements, which offer various outstanding functions, such as gas storage/separation, drug delivery, sensing, and catalysis.^[^ ^11^ ^]^ Despite the bright prospect of MOFs as electrocatalysts, the intrinsic drawbacks such as the low conductivity and the blockage of metal active sites by organic linkers seriously depress their catalytic performances.^[^ ^12^ ^]^ Consequently, a variety of facile and generic synthesis methods, including, but not limited to sonication synthesis,^[^ ^7^, ^13^ ^]^ solvothermal approach,^[^ ^10^, ^14^ ^]^ interface synthesis,^[^ ^15^ ^]^ surfactant‐assisted synthesis,^[^ ^16^ ^]^ chemical vapor deposition,^[^ ^17^ ^]^ template method,^[^ ^18^ ^]^ and exfoliation strategy,^[^ ^19^ ^]^ have been adopted to tailor the morphologies, sizes, and structures of the as‐synthesized MOFs for enhancing their electric conductivity and obtaining more exposed active sites, ultimately promoting the substantial improvement of their comprehensive catalytic performances. Herein, the main synthetic strategies used to manufacture pristine MOF materials are currently classified into two categories, namely, top‐down and bottom‐up methods. Accordingly, the most representative synthesis methods of recently reported pristine MOFs are summarized in **Table** **1**. In the following part, we will highlight the advantages of MOFs in catalyst design, structure–performance relationship exploration, and practical application.

**Table 1:** Summary of the main synthesis methods of pristine MOF materials

Order	Materialsa)	Synthesis methods	Method category	Ref.
1	NiCo‐UMOFNs	Sonication synthesis	Bottom‐up	[7]
2	Co‐TDA	Sonication synthesis	Bottom‐up	[13a]
3	NiFe‐UMNs	Sonication synthesis	Bottom‐up	[13b]
4	LS‐NiFe MOF	Sonication synthesis	Bottom‐up	[13c]
5	PcCu—O_8_—Zn	Solvothermal approach	Bottom‐up	[10]
6	POM‐MOFs	Solvothermal approach	Bottom‐up	[14a]
7	NU‐1000	Solvothermal approach	Bottom‐up	[14b]
8	NENU‐500	Solvothermal approach	Bottom‐up	[14c]
9	D‐Ni‐MOF NSA	Solvothermal approach	Bottom‐up	[14d]
10	CoTCPP‐py‐Cu	Interface synthesis	Bottom‐up	[15a]
11	Ni‐BHT	Interface synthesis	Bottom‐up	[15b]
12	CuBDC‐MOF	Interface synthesis	Bottom‐up	[15c]
13	Zn‐TCPP	Surfactant‐assisted synthesis	Bottom‐up	[16a]
14	Fe‐TPY‐MOL	Surfactant‐assisted synthesis	Bottom‐up	[16b]
15	NH_2_‐MIL‐53(Al)	Surfactant‐assisted synthesis	Bottom‐up	[16c]
16	Zr‐BTB	Surfactant‐assisted synthesis	Bottom‐up	[16d]
17	CTGU‐5 and CTGU‐6	Surfactant‐assisted synthesis	Bottom‐up	[16e]
18	Co—N—C@F127	Surfactant‐assisted synthesis	Bottom‐up	[16f]
19	Fe(TMA)_4_	Chemical vapor deposition	Bottom‐up	[17a]
20	CuBDC and CuCDC	Chemical vapor deposition	Bottom‐up	[17b]
21	ZIF‐A‐LD	Template method	Bottom‐up	[18a]
22	FeCo/CuCo MOF‐74	Template method	Bottom‐up	[18b]
23	MnDMS	Exfoliation strategy	Top‐down	[19a]
24	Zn_2_(bim)_4_	Exfoliation strategy	Top‐down	[19b]
25	Ni_3_(HITP)_2_	Exfoliation strategy	Top‐down	[19c]
26	ZSB‐1	Exfoliation strategy	Top‐down	[19d]
27	Cd‐DP and Zn‐DA	Exfoliation strategy	Top‐down	[19e]
28	Fe(bimCl)_2_	Exfoliation strategy	Top‐down	[19f]
29	STPyP‐Co	Exfoliation strategy	Top‐down	[19g]
30	Cu_2_(CuTCPP)	Exfoliation strategy	Top‐down	[19h]

a) UMOFNs, UMNs = ultrathin MOF nanosheets; TDA = 2,5‐thiophenedicarboxylic acid; LS = lattice‐strained; Pc = phthalocyanine; POM = polyoxometalate; NU‐1000, NENU‐500 = POM‐based MOF; D‐Ni‐MOF NSA = defect‐rich ultrathin Ni(II)‐MOF nanosheet arrays; TCPP = 5,10,15,20‐tetrakis(4‐carboxyphenyl)porphyrinato; py = pyridine; BHT = benzenehexathiol; BDC = 1,4‐benzodicarboxylate; TPY‐MOL = 4′‐(4‐benzoate)‐(2,2′,2″‐terpyridine)‐5,5″‐dicarboxylate‐metal‐organic layers; BTB = 1,3,5‐(4‐carboxylphenyl)‐benzene; CTGU = polymorphic Co‐MOFs; TMA = 1,3,5‐tricarboxylic benzoic acid; CDC = trans‐1,4‐cyclohexanedicarboxylic acid; ZIF‐A‐LD = activated‐ligand‐doped‐ZIF‐8; DMS = 2,2‐dimethylsuccinate; bim = benzimidazole; HITP = 2,3,6,7,10,11‐hexaiminotriphenylene; ZSB =Zn_2_(SBA)_2_(BPTP), (SBA = 4,4′‐sulfonylbibenzoic acid, BPTP = 3,5‐bis(5‐(pyridin‐4‐yl) thiophen‐2‐yl)pyridine)); DP = pyridyl; DA = carboxylic acid; STPyP‐Co = tetra(4‐pyridyl) porphyrin cobalt(II) ultrathin nanosheets; Cu_2_(CuTCPP) = copper(II)‐5,10,15,20‐tetrakis(4‐carboxyphenyl)porphyrin‐Cu(II).

**Figure 2:**
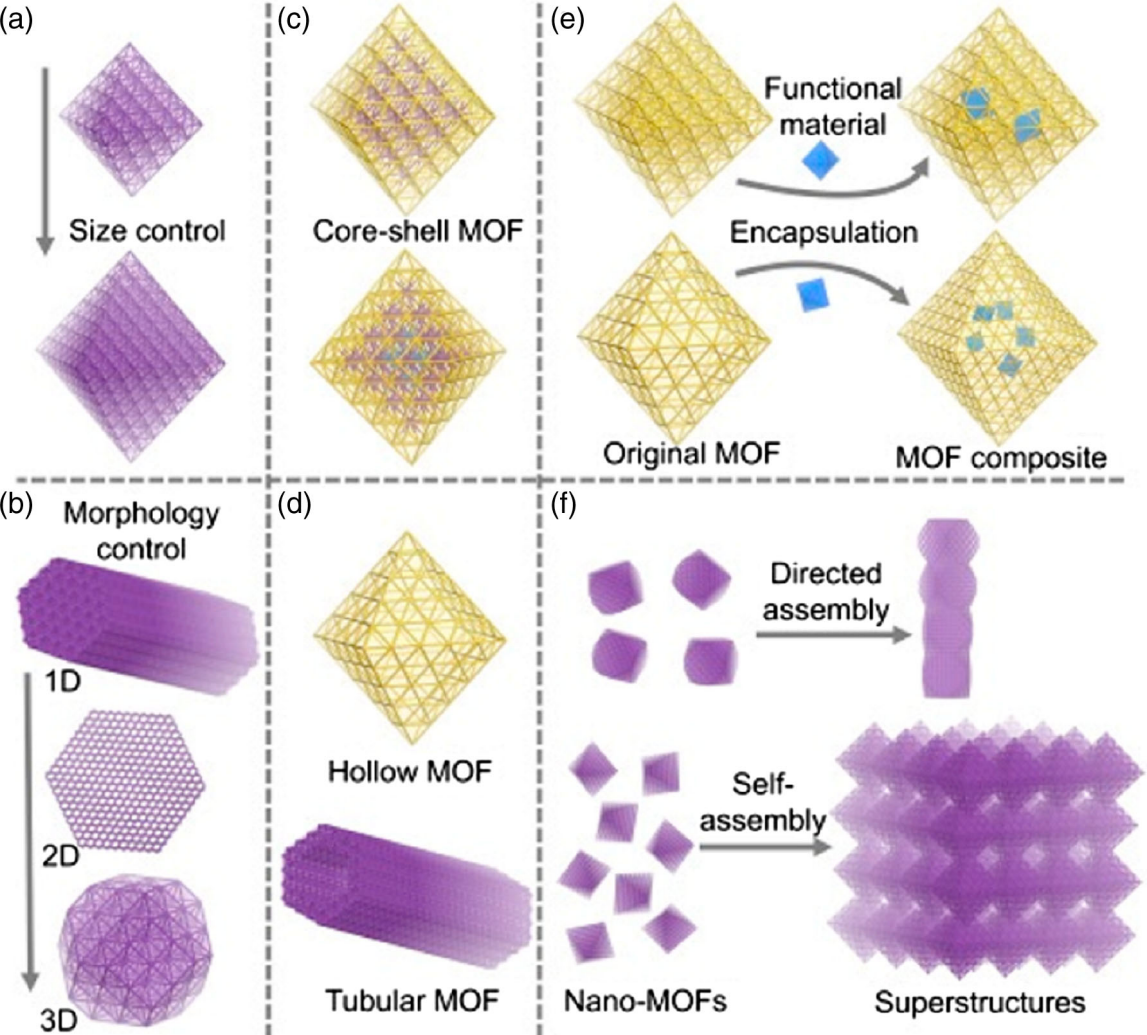
a) Synthesis of MOF nanoparticles with tunable sizes. b) Dimensional control of MOF morphologies from 1D, to 2D, to 3D. c) Construction of core–shell MOFs with different shells. d) Introduction of secondary cavities into MOFs to fabricate hollow and tubular structures. e) Encapsulation of functional materials into MOFs to construct composites. f) Assembly of MOF superstructures by external stimulus or intrinsic driving force. a–f) Reproduced with permission.^[^ ^20^ ^]^ Copyright 2019, Elsevier Inc.

The construction of pristine MOFs with adjustable sizes and governable morphologies (e.g., shapes, dimensions, and hierarchical assemblies) is of great significance in the optimization of their catalytic performances, including activity, selectivity, and stability (**Figure** **2**).^[^ ^20^ ^]^ Specifically, compared with the bulk MOFs, there are abundant disordered regions on the surface of the nanoscale MOFs. The defects or dislocations in the disordered regions provide more active sites for the catalytic reactions. In addition, dimension control is another efficient approach to optimize the catalytic properties of MOFs. Because of the unremitting researchers’ efforts, pristine MOFs with different dimensions from 1D to 3D have been synthesized and applied in the related catalytic reactions.^[^ ^21^ ^]^ Among them, ultrathin 2D MOF nanosheets are becoming an increasingly attractive research hotspot as high‐performance electrocatalysts due to their unique physicochemical properties.^[^ ^22^ ^]^ First, the accessibility of the metals can be greatly improved once the 3D structure of MOFs is converted into a 2D nanosheet. Moreover, it is well established that the 2D structure is conducive to their electron/charge transferability. In addition, the created unsaturated metal sites on the surface of 2D MOFs possess superior catalytic activity and selectivity. In addition, the ultrathin thickness with good permeability facilitates the mass diffusion and transfer during the catalytic processes (see more discussion in Section 3). Therefore, ones are able to achieve targeting design of highly efficient, high‐performance MOFs with atomic precision by paying attention to their morphological concerns.

From a chemistry viewpoint, MOFs are truly fascinating compounds that contain several isomers and various forms. Compared with the traditional catalysts, MOFs constructed by coordination bonds between transition metal (e.g., Ni, Fe, Co, and Mn) ions and organic ligands have exhibited transcending advantages as electrocatalysts. First of all, the high surface area and porosity of MOFs are prerequisites for the mass transport of electrolytes as well as the accessibility between reactants and active sites, which are not realized by metal oxides, hydroxides, or perovskites. Second, the versatile metal centers inside the MOFs are essential to enhance the activity, selectivity, and stability of reactions involving single/multiple electron transfer. Third, the length and surface functional groups of organic ligands also have considerable influences on the catalytic activities and durability of MOFs. For example, the introduction of —NH_2_ or —OH groups into the MOF backbones will provide more favorable binding sites for the intermediates, leading to a high yield.^[^ ^23^ ^]^ Furthermore, the stability of MOFs is seriously affected by the type of ligands. For instance, Dincă and co‐workers recently revealed that the oxidation of Ni species in Ni‐MOF remained unchanged even if the applied potential was up to 1.0 V versus Ag/AgCl.^[19c]^ A phenomenon similar to the oxidation behavior of Ni species in MOFs was also observed in the works reported later.^[^ ^24^ ^]^ Moreover, Dincă and co‐workers designed a series of experiments to explore the role of ligands on the stabilization of MOF‐74.^[^ ^25^ ^]^ In this study, the Mn‐MOF‐74 underwent a severe oxidation process by adding the oxidation reagents (Cl_2_/PhICl_2_). The comprehensive characterizations, including XAS, X‐ray photoelectron spectroscopy (XPS), and magnetic susceptibility measurement, were used to identify the oxidation state of both metal centers and organic ligands. It was found that the chemical valence of metal Mn species remained almost unchanged, whereas the 2,5‐dioxido‐1,4‐benzenedicarboxylate (DOBDC^4−^) ligand was oxidized to its quinoid form, namely, DOBDC^2−^. Such inherent multiformity enabled the rational design of MOFs to target the different electrochemical reactions.

In terms of catalysis, MOFs are ideal candidates for the exploration of structure–performance relationships due to their periodic atomic arrangement and well‐defined structure. Recently, the lattice‐strained NiFe‐MOFs were used as highly active bifunctional electrocatalysts for identifying the active sites and key intermediates during the oxygen redox reaction.^[13c]^ Electrochemical results suggested that the as‐prepared MOFs displayed a mass activity of 500 A g_metal_^−1^ at a half‐wave potential (*E*_1/2_) of 0.83 V versus reversible hydrogen electrode (RHE; all the following mentioned potentials are against RHE) for ORR and 2000 A g_metal_^−1^ at an overpotential of 0.30 V for OER. Moreover, the catalyst could maintain ≈97% of its initial activity after 200 h of continuous ORR/OER reaction in the high current density range of 100–200 mA cm^−2^. The operando synchrotron radiation‐based Fourier transform IR (SR‐FTIR) spectroscopy clearly showed that key superoxide *OOH intermediates appeared on Ni^4+^ sites during ORR and OER, disclosing that a four‐electron redox pathway was responsible for the high electrocatalytic activity of NiFe‐MOFs. Apart from the oxygen redox reaction, Wang and co‐workers reported a generic ligand‐doping approach to obtain an enhanced activity of MOFs for catalyzing CO_2_RR, in which a strong electron‐donating molecule 1,10‐phenanthroline was doped into Zn‐based MOFs, zeolitic imidazolate framework‐8 (ZIF‐8).^[18a]^ Both experimental results and density‐functional theory (DFT) calculation revealed that the electron‐donating nature of phenanthroline played a dominant role in charge transfer. Specifically, it induced the adjacent active sites at the sp^2^ C atoms in the imidazole ligands with more electrons, promoting the generation of *COOH and resulting in an improved activity and a higher Faradaic efficiency for CO (FE_CO_) compared with that of pristine ZIF‐8. Finally, the high selectivity with an FE_CO_ of 90.57% was achieved by mixing the doped ZIFs with a conductive filler.

As for the practical application, it is well established that the major limiting factors are the cost and the performances of catalysts or electrode materials. For instance, even though Ru/Ir‐based materials possess high catalytic activities for OER, their high cost prevents them from being used in industrial‐scale electrolyzers. As a comparison, MOFs are made up of earth‐abundant transition elements (such as Fe, Co, Ni, and Mn), which directly determines their scale‐up application potential. In particular, a growing number of MOFs, such as MOF‐5, HKUST‐1, and MOF‐74, have entered the market at a competitive price. Currently, to produce 1 kg activated MOF‐5 with the formula Zn_4_O(BDC)_3_, where BDC = 1,4‐benzodicarboxylate, 81 L of dimethylformamide (DMF) is required, 1 kg of terephthalic acid and 3.5 kg of zinc acetate dihydrate with 63% yield, and these raw materials cost 414.6, 34.0, and 78.7 USD, respectively. Hence, its total cost is roughly 527 USD kg^−1^, which is much lower than that of equivalent Ru or Ir.^[^ ^26^ ^]^ Armed with superior catalytic performances, MOFs are becoming the favored choice for mass production and practical application to replace the expensive commercial noble metals.

## 3 Recent Progress on Pristine MOFs for Electrocatalysis

### 3.1 ORR

**Figure 3:**
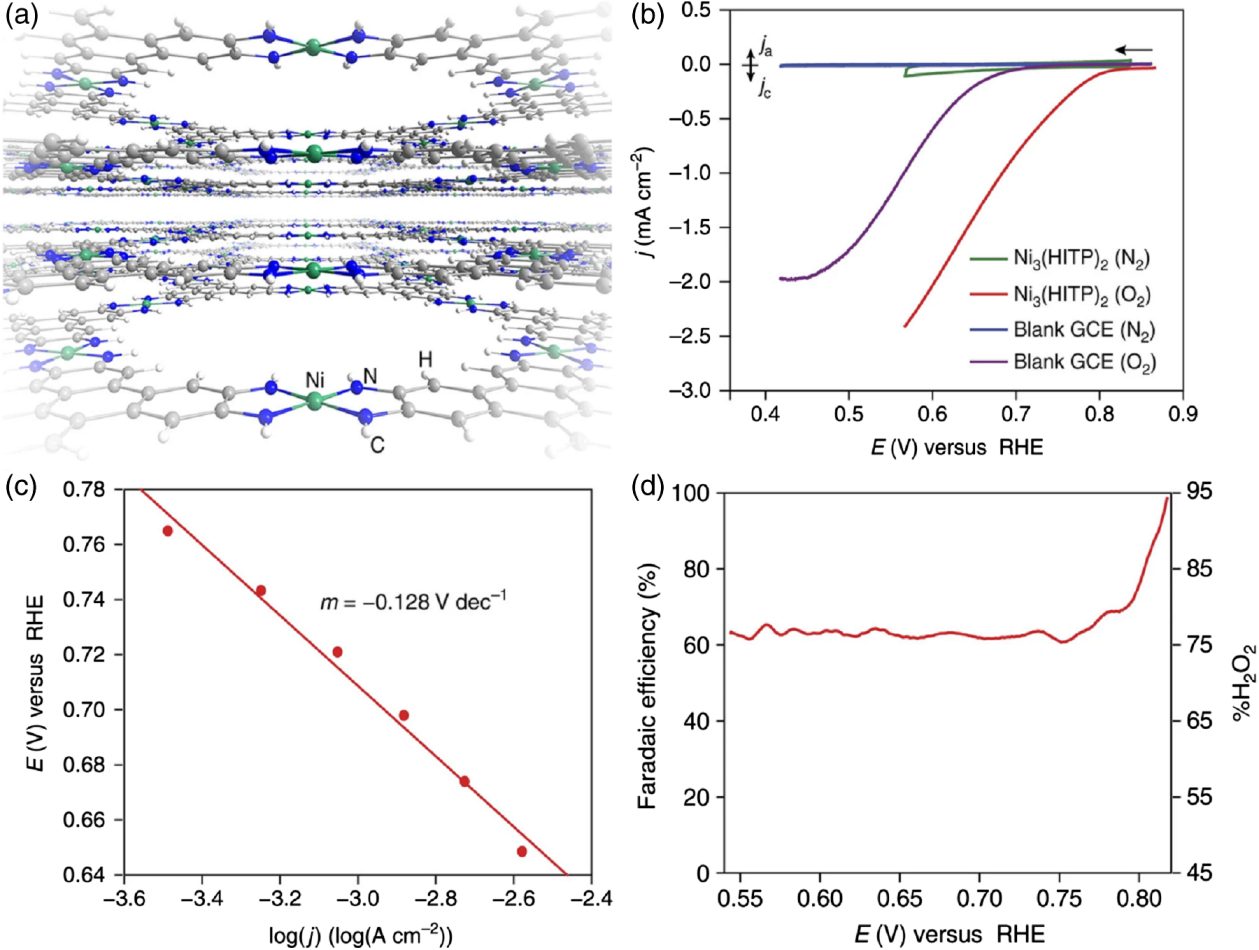
a) Perspective view of the 2D‐layered structure of Ni_3_(HITP)_2_. b) Polarization curves of Ni_3_(HITP)_2_ and the blank glassy carbon electrode under N_2_ versus O_2_ atmosphere in 0.1 m KOH electrolyte at a scan rate of 5 mV s^−1^. c) Activation‐controlled Tafel plot for the Ni_3_(HITP)_2_‐electrocatalyzed ORR. d) Potential‐dependent FE of Ni_3_(HITP)_2_ for H_2_O_2_ production and %H_2_O_2_ production during the ORR at a pH of 13. a–d) Reproduced under the terms of the CC‐BY 4.0 license.^[^ ^30^ ^]^ Copyright 2016, The Authors, published by Springer Nature.

The cathodic ORR involving a four‐electron transfer is the rate‐determining step (RDS) in various sustainable and efficient energy conversion techniques, including fuel cells and metal–air batteries owing to its sluggish kinetics.^[^ ^27^ ^]^ Presently, carbon‐supported Pt nanoparticles (Pt/C) have been considered as the commonly used ORR catalysts with excellent electrocatalytic performances.^[^ ^28^ ^]^ Nevertheless, the scarcity and high cost of Pt as well as the poor anti‐methanol poisoning ability of Pt/C largely impede their commercial application. Enormous efforts have been made to create low‐cost high‐performance MOF‐based electrocatalysts for ORR.^[^ ^29^ ^]^ As an example, a 2D Ni_3_(HITP)_2_ MOF (HITP = 2,3,6,7,10,11‐hexaiminotriphenylene) with a decent electrical conductivity of *σ* = 40 S cm^−1^ was successfully synthesized and used as the electrocatalyst for ORR (**Figure** **3**).^[^ ^30^ ^]^ Remarkably, Ni_3_(HITP)_2_ MOF presented a positive *E*_onset_ of 0.82 V and a small Tafel slope of −128 mV dec^−1^ in 0.1 m KOH electrolyte, comparable to those of the most active non‐Pt group metal (nPGM) electrocatalysts to date. Various characterization results and electrochemical data uncovered that the high crystallinity of MOFs, excellent electrical conductivity, and facile accessibility of molecular species enabled MOFs outstanding electrocatalytic performances in fuel cells and electrolyzers.

**Figure 4:**
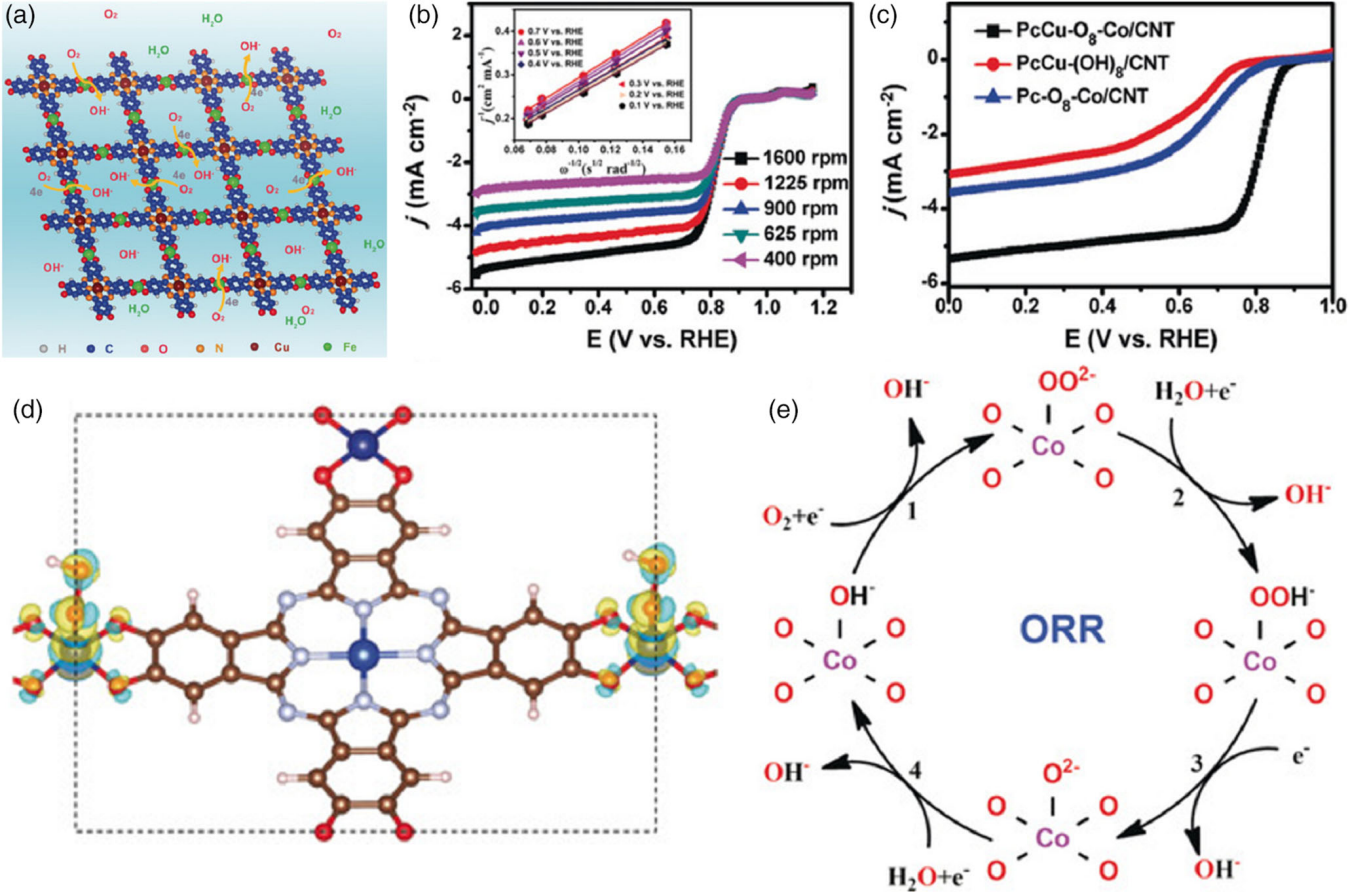
a) Schematic structure of PcCu—O_8_—M. b) ORR polarization curves of PcCu—O_8_—Co/CNT at different rotating speeds with the corresponding *K*–*L* plots of PcCu—O_8_—Co/CNT (inset). c) ORR polarization curves of PcCu—(OH)_8_/CNT, Pc—O_8_—Co/CNT, and PcCu—O_8_—Co/CNT. d) The differential charge density image of PcCu—O_8_—Co with OOH^−^ intermediates on the Co—O sites (red: O; brown: C; light blue: Cu; dark blue: Co; white: H; gray: N, and the isosurface value is 0.001 e Å^−3^). e) Proposed reaction mechanism for the ORR. a–e) Reproduced with permission.^[^ ^31^ ^]^ Copyright 2019, Wiley‐VCH.

More recently, Feng and co‐workers proposed a simple solvothermal method to synthesize the copper phthalocyanine‐based 2D conjugated MOFs with square‐planar cobalt bis(dihydroxy) complexes (Co—O_4_) as linkages (PcCu—O_8_—Co) and layer‐stacked structures for efficient ORR (**Figure** **4**).^[^ ^31^ ^]^ A highly crystalline structure and optimized spin state of the cobalt centers in PcCu—O_8_—Co were obtained, where the unpaired electron in the σ* antibonding orbital (*e*_g_ = 1) in the cobalt node brought about the remarkable ORR activity of Co—O_4_ centers.^[16f]^ Notably, the resultant PcCu—O_8_—Co MOF mixed with carbon nanotubes shows an excellent catalytic ORR performance (*E*_1/2_ = 0.83 V, *n* = 3.93, and *j*_L_ = 5.3 mA cm^−2^) in alkaline media, which was the record‐high value among the previously reported MOF‐based catalysts. The excellent activity of the as‐prepared electrocatalyst was attributed to the high coverage of electrochemically active Co sites, outstanding conductivity, and a highly ordered porous structure. Furthermore, in situ Raman spectro‐electrochemistry and theoretical modeling as well as contrast catalytic tests not only illustrated that the cobalt nodes could act as the ORR active centers but also validated a proposed ORR mechanism based on the derived results. When used as a cathode catalyst in a zinc–air battery, the PcCu—O_8_—Co MOF exhibited an extraordinary discharge performance and capacity with a maximum power density of 94 mW cm^−2^, even outperforming the state‐of‐the‐art Pt/C catalyst (78.3 mW cm^−2^). In 2020, Yin and co‐workers creatively introduced a facile low‐temperature iodine treatment (at 170 °C without the participation of any solvents and inert atmospheres) to synthesize a modified pristine MOF (I_2_&ZIF‐67) composed of Co^2+^ and 2‐methylimidazole as the metal node and organic linker, respectively.^[^ ^32^ ^]^ The resulting I_2_&ZIF‐67 catalyst displayed a significantly enhanced catalytic ORR property comparable to that of 20% Pt/C in basic media in terms of its *E*_1/2_ (≈0.83 V) and Tafel slope (≈37.3 mV dec^−1^) as well as excellent durability, which was primarily attributed to the greater number of exposed active species (i.e., pyridinic‐N, Co^2+^, and I^3−^) on the MOF surface induced by the strong interplay between I_2_ and ZIF‐67. These works suggested that optimizing both the architecture and electronic structure of MOFs indeed played a critical role in the development of highly efficient MOF‐based catalysts for ORR.

In addition to the pristine MOF‐based monofunctional ORR electrocatalyst, NiFe‐naphthalenedicarboxylate (NDC) MOF nanosheet arrays on Ni foam were constructed by a hydrothermal method with strain engineering, serving as highly active bifunctional electrocatalysts for catalyzing both ORR and OER.^[13c]^ The lattice‐strained pristine MOFs delivered a mass activity of 500 A g_metal_^−1^ at a high *E*_1/2_ of 0.83 V for ORR and 2000 A g_metal_^−1^ at an overpotential of 0.30 V for OER with decent stability after 200 h. Subsequently, SR‐FTIR spectroscopy together with XAS techniques confirmed that key superoxide *OOH intermediates appeared on Ni^4+^ sites during ORR and OER, further revealing a 4e^−^ redox reaction mechanism of high‐performance NiFe‐MOFs.

### 3.2 HOR

**Figure 5:**
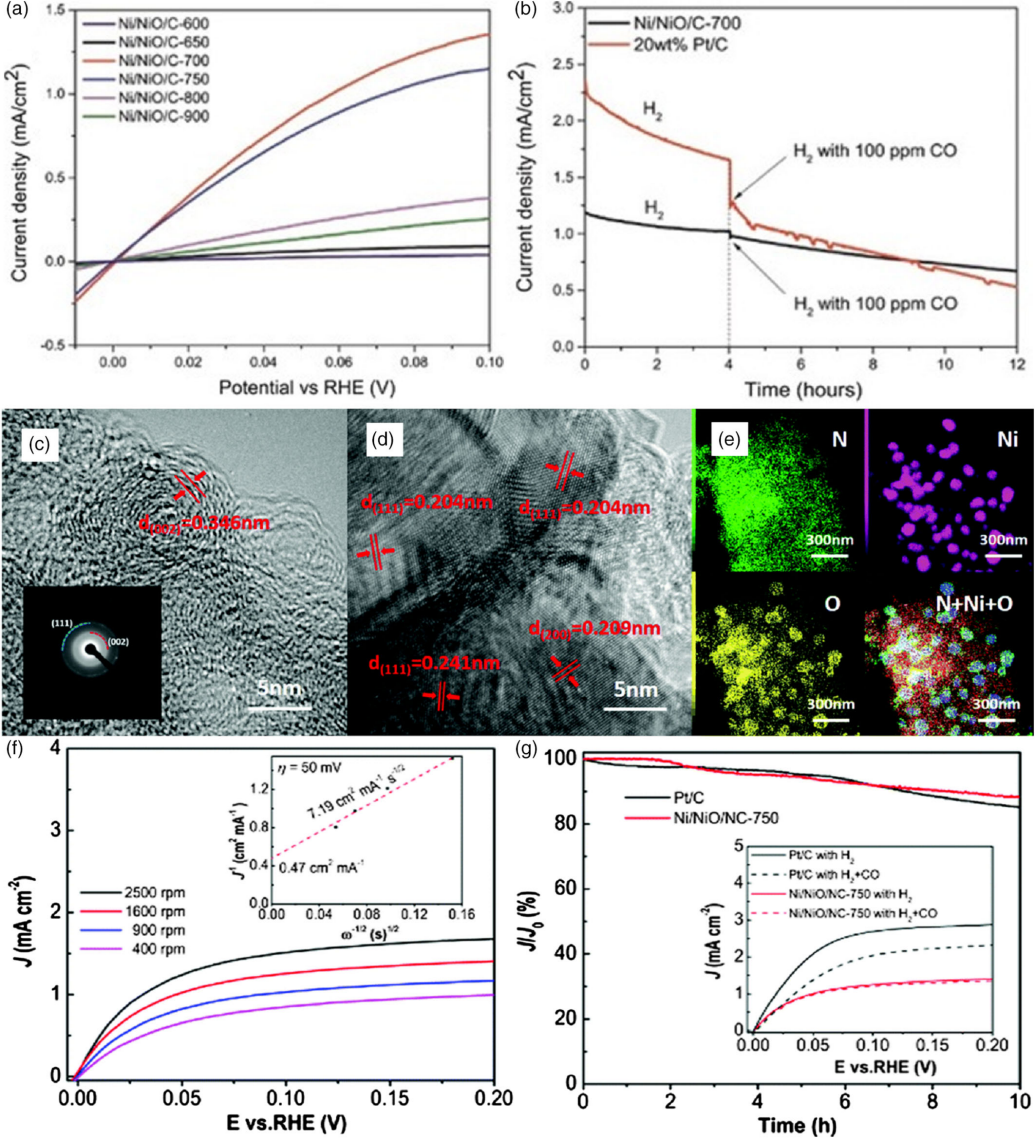
a) Polarization curves of Ni‐MOF‐based samples for HOR in 0.1 m KOH at 1600 rpm with a scan rate of 1 mV s^−1^. b) Chronoamperometry curves of Ni/NiO/C‐700 and Pt/C before and after the addition of 100 ppm CO. Reproduced with permission.^[^ ^36^ ^]^ Copyright 2019, Wiley‐VCH. c,d) HRTEM images of Ni/NiO/NC‐750 (inset: selected‐area electron diffraction pattern). e) Energy‐dispersive X‐ray spectroscopy element mapping of different elements. f) Polarization curves of Ni/NiO/NC‐750 at different rotation rates (inset: Koutecky–Levich plot). g) Chronoamperometry curves (inset: polarization curves in H_2_‐ or H_2_ + CO‐saturated 0.1 m KOH). a–g) Reproduced with permission.^[^ ^38^ ^]^ Copyright 2021, The Royal Society of Chemistry and the Chinese Chemical Society.

Apart from the most commonly used proton‐exchange membrane fuel cells (PEMFCs), anion‐exchange membrane fuel cells (AEMFCs) have been regarded as the other category of low‐temperature fuel cells using H_2_ as a clean fuel, especially hydroxide exchange membrane fuel cell (HEMFC), where the HOR occurring at the anode and the cathodic ORR are two fundamental half‐cell reactions.^[^ ^33^ ^]^ Although massive advancement of both anion‐conductive membranes and PGM catalysts allow HEMFC to become an encouraging alternative to PEMFC, the low kinetics of the HOR process in alkaline media is still a significant bottleneck for its practical application, causing around two orders of magnitude reduction of PGM catalysts’ catalytic HOR activities when transferred from acidic electrolytes to basic environments.^[^ ^34^ ^]^ Also, the high price of PGM catalysts is another big concern, with ≈80% cost on the cathode side and the rest on the cathode side.^[^ ^35^ ^]^ Hence, seeking high‐performance, low‐cost, and earth‐abundant HOR electrocatalysts for HEMFCs is highly desired to minimize Pt utilization. Making use of the modular nature and decent controllability of MOFs, pristine MOF‐based materials stand out from a variety of HOR catalysts. For example, Sun and co‐workers creatively reported a Ni‐based MOF (Ni‐benzene‐1,3,5‐tricarboxylic acid [BTC]) transformed to a cost‐effective Ni/NiO/C electrocatalyst with abundant Ni/NiO interfacial sites for HOR in alkaline media (**Figure** **5**a,b).^[^ ^36^ ^]^ The as‐fabricated MOF‐based material exhibited a much‐boosted electrocatalytic HOR performance, which was firmly evidenced in terms of its *E*_onset_ of nearly 0, superior anti‐carbon‐monoxide poisoning ability, and long‐term stability compared with commercial Pt/C, thus manifesting its super‐high likelihood of long service in practical HEMFCs. Furthermore, experimental analyses coupled with DFT simulations unveiled the foremost role of both interfacing Ni/NiO and highly conductive graphene layers in optimizing electronic and aerobic properties for ultimately promoting the HOR process.

A “volcano” relationship between the binding energy of hydrogen and the HOR activity strongly proves that the adsorbed hydrogen is a key HOR intermediate.^[^ ^37^ ^]^ In consequence, skillfully adjusting hydrogen adsorption will be a powerful strategy to accelerate HOR electrocatalysis of MOFs. In this respect, a highly porous Ni‐MOF‐based catalytic material (Ni/NiO/NC) composed of Ni^2+^ metal sites and 2‐methylimidazole ligands was developed most recently (in 2021) and then probed for its bifunction for both HOR and ORR catalysis under an alkaline environment (Figure 5c–g).^[^ ^38^ ^]^ Benefiting from its favorable composition and well‐defined heterostructures, this electrocatalyst showed a high mass activity of 9.09 mA mg_Ni_^−1^ at an overpotential of 50 mV, outperforming the previously reported Ni‐based catalysts for the 2e^−^ transfer HOR process.^[^ ^36^, ^39^ ^]^ Also, it possessed better robustness with lower anodic current decay (88.4%) than that of commercial Pt/C (85.3%) after 10 h immersion in H_2_‐saturated 0.1 m KOH solutions at 0.1 V. Specifically, the heterogeneous Ni—NiO interfaces with oxygen vacancies brought about the enhanced adsorption strength of hydrogen and hydroxyl for HOR due to the markedly modified electronic states; meanwhile, the abundant pyridinic‐N functional groups acted as prime active sites for ORR. This work rendered such novel material a promising electrode catalyst with inherent characteristics of MOFs for both HOR and ORR in HEMFCs.

### 3.3 HER

**Figure 6:**
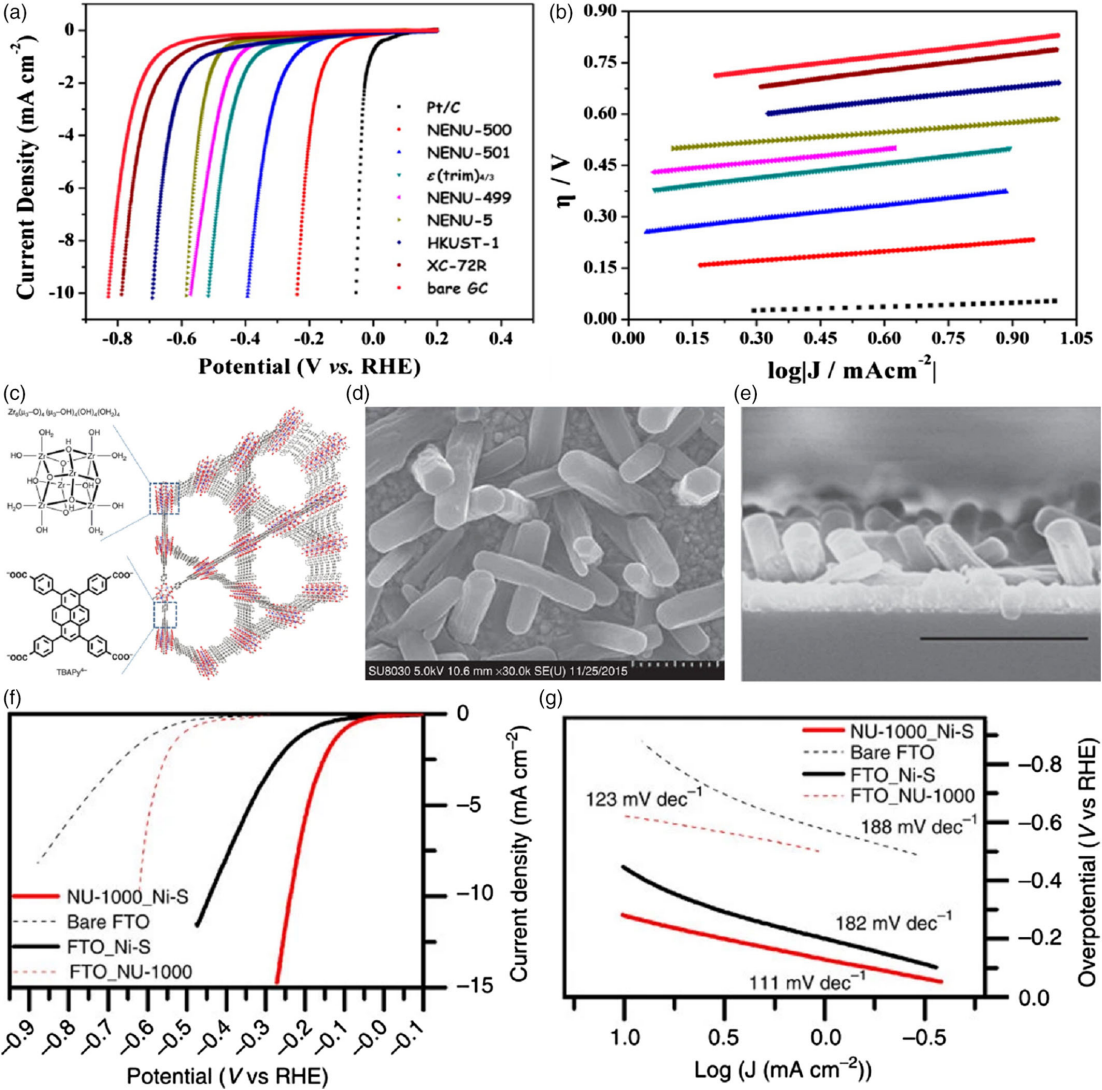
a) Polarization curves of the prepared catalysts in 0.5 m H_2_SO_4_ solution. b) The corresponding Tafel plots of the prepared catalysts. a,b) Reproduced with permission.^[14c]^ Copyright 2015, American Chemical Society. c) Schematic representation of crystal structure of NU‐1000. d) scanning electron microscopy (SEM) image of NU‐1000_Ni—S film (scale bar: 1 μm). e) Cross‐sectional SEM image of NU‐1000_Ni—S film (scale bar: 2 μm). f) *J*–*V* curves of bare fluorine‐doped tin oxide (FTO), FTO_NU‐1000, FTO_Ni—S, and NU‐1000_Ni—S electrodes. g) The corresponding Tafel plots of these four electrodes. c–g) Reproduced under the terms of the CC‐BY 4.0 license.^[14b]^ Copyright 2015, The Authors, published by Springer Nature.

Electrocatalytic water splitting has long been recognized as one of the most promising routes to generate sustainable hydrogen energy from water.^[^ ^40^ ^]^ Although the Pt‐based catalysts are known to be the most kinetically favorable electrocatalytic materials for HER, their scarcity and high cost greatly restrict their large‐scale use in actual electrolyzers.^[^ ^41^ ^]^ Among the numerous works of replacing Pt‐based electrodes with nPGM catalysts for HER, MOFs have shown the superiority. For instance, a series of polyoxometalate (POM)‐based MOFs containing monomeric, dimeric, or chain‐like Zn‐ϵ‐Keggin building blocks connected by trimesate ions were fabricated as electrocatalysts for HER.^[14a]^ The optimized POM‐based MOF displayed a remarkable electrocatalytic HER activity with a yield higher than 95% and a turnover number (TON) of 1.2 × 10^5^ after 5 h. The computational analysis indicated that the organic ligands were crucial for regulating the surface charge of cations in MOFs and, thus, tuning the adsorption strength of the intermediates. To further gain the accessibility of metal sites, the POM‐based NENU‐500 MOF, [TBA]_3_[ϵ‐PMo_8_Mo_4_O_36_(OH)_4_Zn_4_][BTB]_4/3_ (BTB = benzene tribenzoate and TBA^+^ = tetrabutylammonium ion), with a 3D open framework was designed for electrochemically catalyzing the HER process.^[14c]^ Impressively, the as‐prepared NENU‐500 MOF with sufficient exposed active sites afforded a high catalytic HER activity in acidic media with a small Tafel slope of 96 mV dec^−1^, an *E*_onset_ of 180 mV, and a current density of 10 mA cm^−2^ at a relatively low overpotential of 237 mV (**Figure** **6**a,b). Moreover, it exhibited not only decent stability in the air but also excellent tolerance in both acidic and basic media. In another study, Hod et al. synthesized a Zr‐based NU‐1000 MOF thin film, [Zr_6_O_16_H_16_][TBAPy]_2_ (TBAPy = 1,3,6,8‐tetrakis(*p*‐benzoic acid)pyrene), electrodeposited with a layer of Ni—S, achieving a superior electrocatalytic HER activity (Figure 6c–g).^[14b]^ The kinetic overpotential for HER on the NU‐1000 electrode was ≈210 mV lower than that of pristine electrodeposited Ni—S under identical conditions. The excellent activity was attributed to the facilitated proton transport and the improved immediate chemical environment caused by NU‐1000 MOF during the HER electrocatalysis.

**Figure 7:**
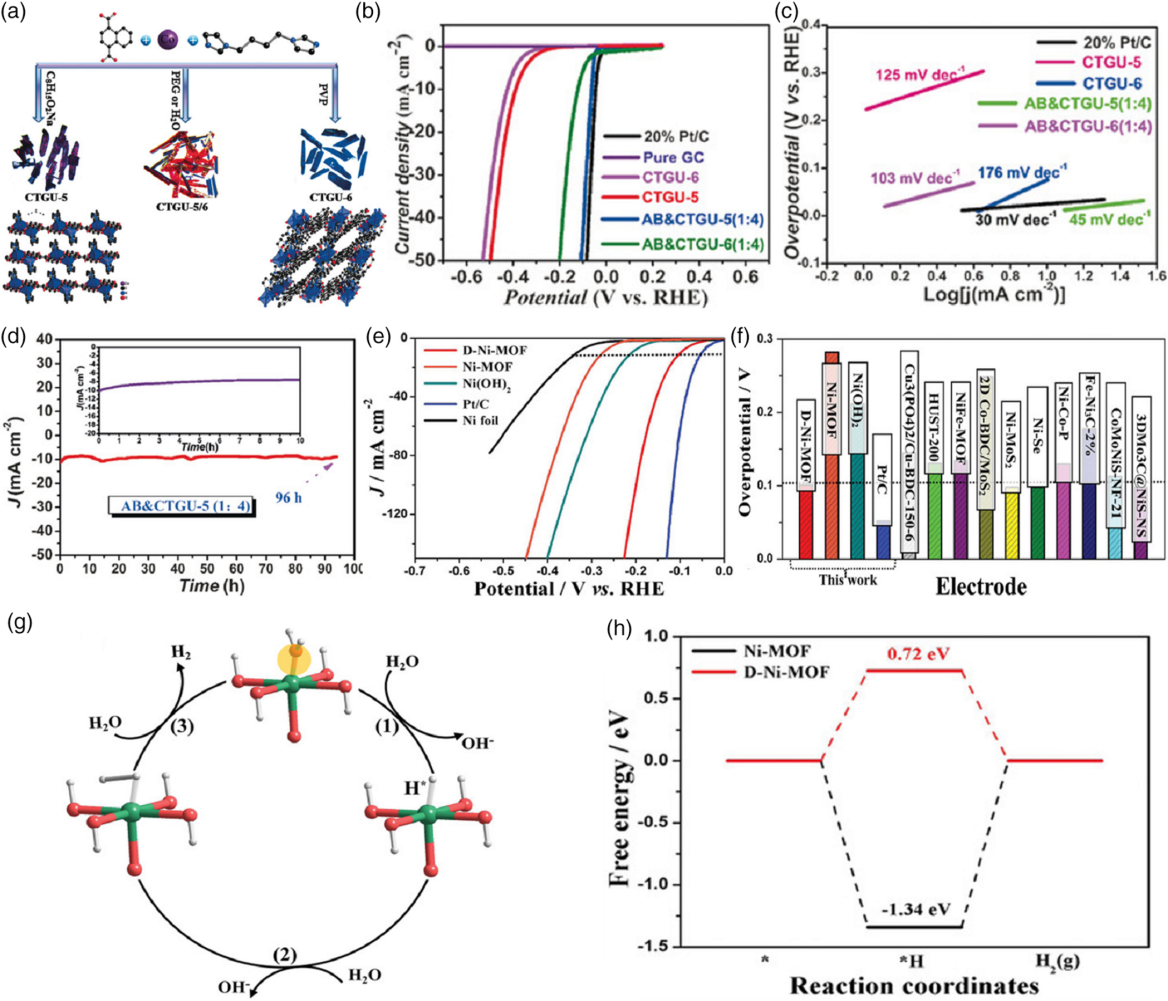
a) Schematic representation of two isomeric Co‐MOFs and their crystal structure diagrams. b) Polarization curves for various catalysts in 0.5 m H_2_SO_4_ electrolyte. c) Tafel plots obtained from the corresponding polarization curves. d) Chronoamperometric profiles of AB&CTGU‐5(1:4) at −0.255 V. a–d) Reproduced with permission.^[16e]^ Copyright 2017, Wiley‐VCH. e) Linear sweep voltammetry plots of defect‐rich ultrathin Ni(II)‐MOF nanosheet arrays (D‐Ni‐MOF) and other catalysts for the HER. f) Comparison of typical catalysts’ overpotentials at 10 mA cm^−2^ in 1 m KOH solution. g) The proposed electrocatalytic mechanism of D‐Ni‐MOF. h) Calculated free‐energy diagram in the pristine Ni‐MOF and D‐Ni‐MOF at 0 V. e–h) Reproduced with permission.^[14d]^ Copyright 2020, Wiley‐VCH.

Apart from the metal nodes, the organic linkers also exert significant impacts on the coordination configuration and porous network of MOF‐based catalysts, subsequently leading to the modulated catalytic performances. Two Co‐MOFs, CTGU‐5 and CTGU‐6, were synthesized by mixing Co(ClO_4_)_2_, naphthalene‐1,4‐dicarboxylic acid, and 1,4‐bis(imidazole)butane followed by selective crystallization into pure 2D or 3D net using an anionic or neutral surfactant (**Figure** **7**a–d).^[16e]^ Despite the similar coordination mode by Co^II^ to ligands, the H_2_O molecule in these two MOFs differed noticeably in its bonding to the framework, which, in turn, affected the crystal structures and electrocatalytic performances for HER. Both experimental and computational studies proved that 2D CTGU‐5 with the coordinated water possessed a superior electrocatalytic activity compared with that of 3D CTGU‐6 with the lattice water. Notably, the composite material of acetylene black (AB) integrated with CTGU‐5 yielded the best HER performance among the reported MOFs with a positive *E*_onset_ of 18 mV, a small Tafel slope of 45 mV dec^−1^, and a high exchange current density of 0.86 mA cm^−2^, as well as long‐term durability. More recently, Li and co‐workers put forward a simple alkali‐etched strategy to synthesize defect‐rich ultrathin Ni(II)‐MOF nanosheet arrays (denoted as Ni_2_(OH)_2_(BDC)) for efficiently catalyzing HER in a 1 m KOH electrolyte (Figure 7e–h).^[14d]^ Interestingly, the as‐fabricated MOF nanosheet arrays showed an exceptional electrocatalytic activity for producing H_2_ with a much lower overpotential and a smaller Tafel slope (101 mV at 10 mA cm^−2^ and 50.9 mV dec^−1^) than those of defect‐free Ni‐MOF nanosheet arrays (232 mV and 85.6 mV dec^−1^), Ni(OH)_2_ nanosheet arrays (282 mV and 111.4 mV dec^−1^), and the currently reported works. The mechanism analysis illustrated that the open metal sites with high valence states and oxygen vacancies on the metal‐oxide layers played a favorable role in reducing the rate‐determining energy barrier in HER. Simultaneously, the 3d projected density of states (PDOS) revealed that defect‐rich MOF nanosheet arrays nearly displayed a metallic phase property owing to the incorporation of K^+^, suggesting its better electrical conductivity. The work highlighted that the creation of defects and the improvement of electrical conductivity were vital to tailor the catalytic properties of MOF‐based catalysts.

### 3.4 OER

OER at the anode involving a four electrons transfer is the key step that limits the energy conversion efficiency of many clean energy devices, such as water electrolyzers and rechargeable metal–air batteries.^[^ ^42^ ^]^ Ru‐ or Ir‐based catalysts are regarded as the most efficient OER catalysts so far; however, the prohibitive cost and scarcity severely hinder their widespread implementation. In the past few decades, tremendous efforts have been devoted to the development of various nanomaterials (such as metal oxides, hydroxides, and perovskites) to replace noble metal‐based ones.^[^ ^43^ ^]^ Among them, MOFs are the ideal platforms for constructing these efficient OER electrocatalysts.^[^ ^44^ ^]^ First, different from traditional‐doped or compound catalysts, the metal tunability and periodic element arrays of MOFs allow us to optimize the activities of bimetallic or multimetallic catalysts at a precisely controllable atomic level, which is extremely beneficial to promote the OER catalytic activity via electronic coupling.^[^ ^45^ ^]^ Meanwhile, the periodic structure of MOFs provides an explicit molecular mode to explore the exact structure–performance relationship at the atomic or molecular level in various electrocatalytic processes.^[^ ^46^ ^]^ In addition, compared with traditional porous materials such as zeolites confined by rigid tetrahedral oxide skeletons, the porosity and flexibility of MOFs are unique origins of the durability of OER catalysts.^[^ ^7^, ^47^ ^]^

**Figure 8:**
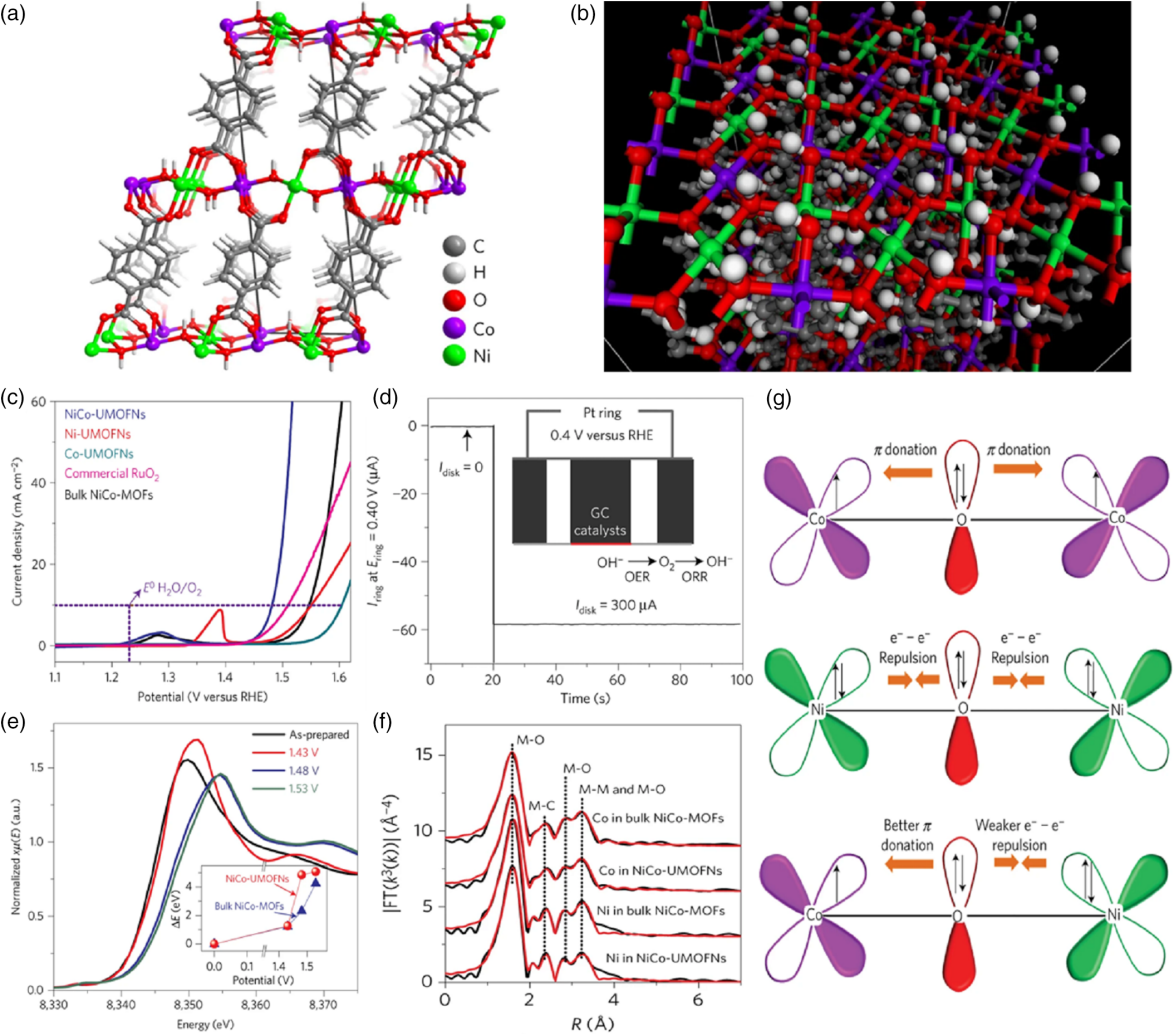
a) Crystal structure of NiCo‐UMOFNs. b) Simulation image of the (200) lattice plane of a UMOFNs surface. c) Polarization curves of various MOFs and RuO_2_ in O_2_‐saturated 1 m KOH solution. d) FE testing of NiCo‐UMOFNs using the rotating ring‐disk electrode technique with testing mechanism (inset). e) Comparison of Ni K‐edge XANES data of NiCo‐UMOFNs with the relative energy shifts of the main absorption peaks (inset). f) Ex situ EXAFS data in *R*‐space and the best‐fit results. g) Schematic representation of the electronic coupling between Co and Ni in UMOFNs. a–g) Reproduced with permission.^[^ ^7^ ^]^ Copyright 2016, Springer Nature.

In 2016, a class of UMOFNs, CoNi(OH)_2_(BDC), were synthesized through a facile ambient temperature sonication synthesis method and then applied for electrocatalytic OER under alkaline conditions (**Figure** **8**).^[^ ^7^ ^]^ NiCo‐UMOFNs loaded on Cu foam exhibited a low *E*_onset_ of 1.39 V and a relatively small overpotential of ≈189 mV at 10 mA cm^−2^ in an O_2_‐saturated 1 m KOH solution. Meanwhile, a highly stable current density (only 2.6% decay) was observed at a constant overpotential of 0.25 V and maintained for at least 200 h, demonstrating an excellent electrocatalytic OER activity and corresponding long‐term catalytic stability of NiCo‐UMOFNs catalysts. A combination of XAS analysis and DFT calculation disclosed that the coordinatively unsaturated metal atoms in the ordered structure of MOFs were the active centers for OER, and the interaction between Ni and Co metal atoms further exerted decisive impacts on the regulation of electrocatalytic performances. Therefore, ultrathinning MOFs provided an advanced platform toward developing highly active heterogeneous catalysts with atomic precision.

**Figure 9:**
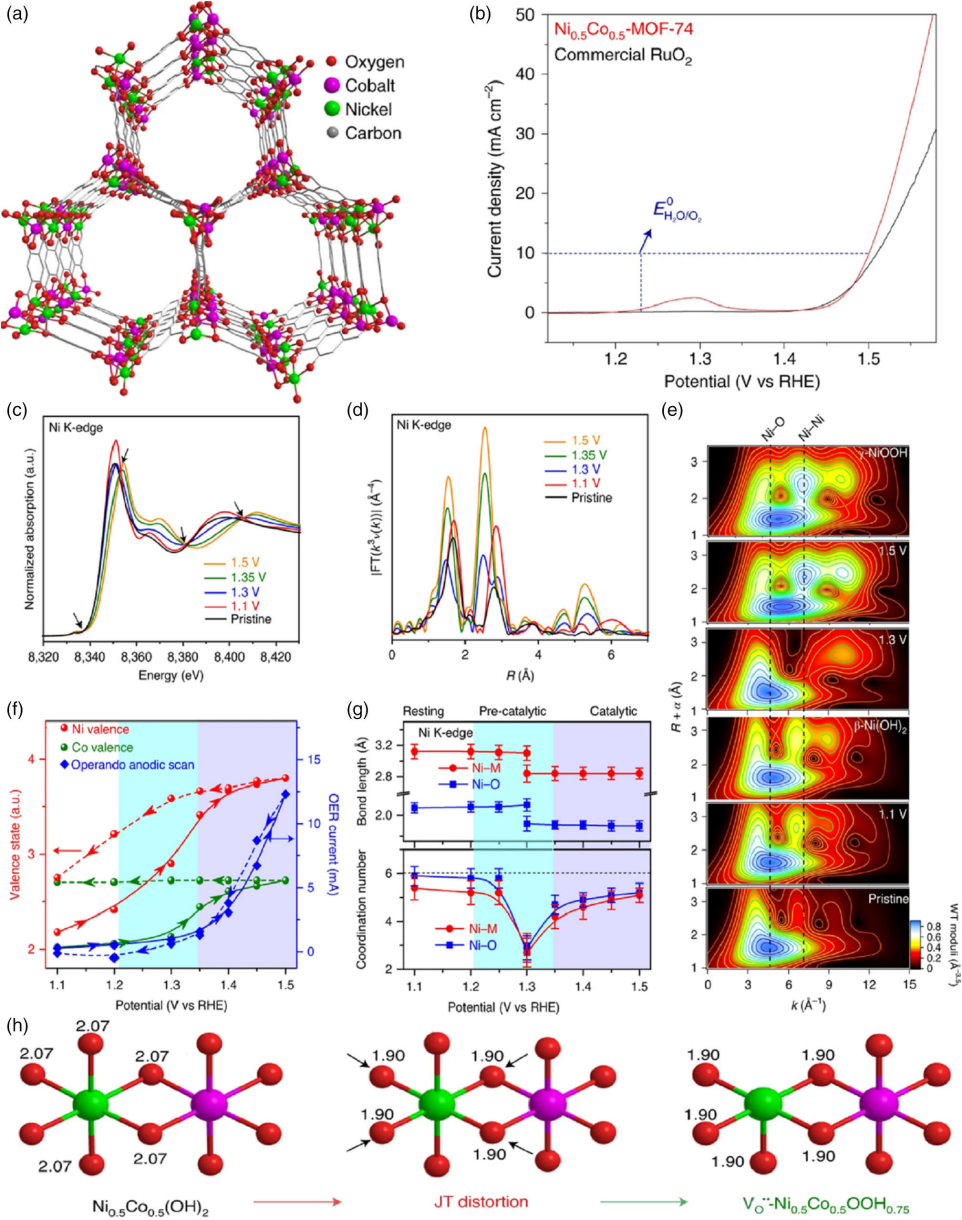
a) Crystal structure of Ni_0.5_Co_0.5_‐MOF‐74. b) Polarization curves of Ni_0.5_Co_0.5_‐MOF‐74 and RuO_2_ in O_2_‐saturated 1 m KOH solution. c) Ni K‐edge XANES spectra recorded at different potentials. d) The corresponding Fourier‐transformed *k* ^3^‐weighted EXAFS signals. e) Comparison of Ni K‐edge EXAFS wavelet transforms (WTs) recorded at 1.1, 1.3, and 1.5 V with an optimum resolution at 3.0 Å. f) Change in the Ni and Co valence states and the OER current as a function of applied potential. g) Changes of bond length and coordination number for the Ni—O and Ni—M coordination shells. h) Structure models of the theoretical spectra (green: Ni; purple: Co; red: O) with the bond lengths of M—O, in Å. a–h) Reproduced with permission.^[^ ^47^ ^]^ Copyright 2020, Springer Nature.

To investigate the fundamental issues, such as atomic‐level activation mechanism, reversibility, and the essence of synergistic effects in bimetallic systems, the well‐known and widely used MOF‐74 series (CoNi(DHTP)(H_2_O)_2_·8H_2_O; DHTP = 2,5‐dihydroxyterephthalate) were selected as the model OER catalysts (**Figure** **9**).^[^ ^47^ ^]^ Both operando XAS and high‐resolution transmission electron microscopy (HRTEM) techniques were used to probe the structural transformation occurring at metal nodes of MOF‐74. Noteworthily, there was a two‐phase structural transition found at metal nodes with Ni_0.5_Co_0.5_(OH)_2_ and Ni_0.5_Co_0.5_OOH_0.75_ forming at relatively low and high applied potentials, respectively, of which the Ni_0.5_Co_0.5_OOH_0.75_ formed in situ with abundant oxygen vacancies and high oxidation states was responsible for the extraordinary OER activity. Moreover, adjusting the ratio between Ni and Co in the NiCo‐MOF‐74 series was able to tune this potential‐induced two‐step structural reconstruction by altering the geometric and electronic structures of active Ni—O and Co—O moieties, which, in turn, resulted in the huge improvement of catalytic activity. Accordingly, a Ni_0.9_Fe_0.1_‐MOF was fabricated in line with the principle of synchronous structural transformation, which merely required the low overpotentials of 198 and 231 mV to achieve 10 and 20 mA cm^−2^ toward OER, respectively. Encouragingly, these findings provided some critical insights into both the understanding of the fundamental processes of structural transformation during the OER and the rational design of high‐performance pristine MOF‐based OER catalysts.

Aside from the breakthrough of bimetallic systems, the metal adjustability and periodic element arrays of pristine MOFs open the door for their trimetallic catalysts to greatly facilitate the OER process. For instance, Zhang and co‐workers, for the first time, used a mild one‐pot solution‐phase method to develop hierarchical trimetallic NiCoFe‐based MOF nanofoams at ambient temperature with controlled molar ratios, where metal acetate and 1,4‐bezenedicarboxylate acid (1,4‐BDC) were used as precursors and organic ligand, respectively.^[^ ^48^ ^]^ Interestingly, the resulting MOF nanostructures could directly serve for OER electrocatalysis with an extremely low overpotential of 257 mV at 10 mA cm^−2^, a small Tafel slope of 41.3 mV dec^−1^, and long‐term durability in a 1 m KOH solution. This can be clearly explained by the existence of electrochemically transformed metal hydroxides and oxyhydroxides—active catalytic species. This work inspired the related MOF research community with novel hierarchical foam‐like MOF architecture, which provided an effective platform toward the tunability of MOFs’ morphologies and compositions.

### 3.5 CO_2_RR

In recent years, the concentration of CO_2_ in the atmosphere has been experiencing an increasingly rapid rise, gradually leading to undesired climate change (e.g., ocean acidification and global warming).^[^ ^49^ ^]^ Hence, converting carbon dioxide into various high value‐added organic fuel molecules (e.g., CO, CH_3_OH, CH_4_, HCOOH, etc.) via effective electrochemical technologies has been considered as a promising strategy to recycle and utilize this abundant and inexpensive carbon resource.^[^ ^50^ ^]^ Furthermore, the electrocatalytic CO_2_RR that takes place at room temperature and under ambient pressure holds enormous promise from both economic and environmental perspectives.^[^ ^51^ ^]^ However, due to the thermodynamically stable and kinetically inert nature of CO_2_, as well as the competition with HER in aqueous electrolytes, highly active and selective electrocatalysts are particularly significant in this endeavor. In recent years, some pristine MOF‐based catalysts have been extensively investigated to electrochemically catalyze the CO_2_RR process, which can outperform many noble metal‐based catalysts.[^18^, ^19^]

**Figure 10:**
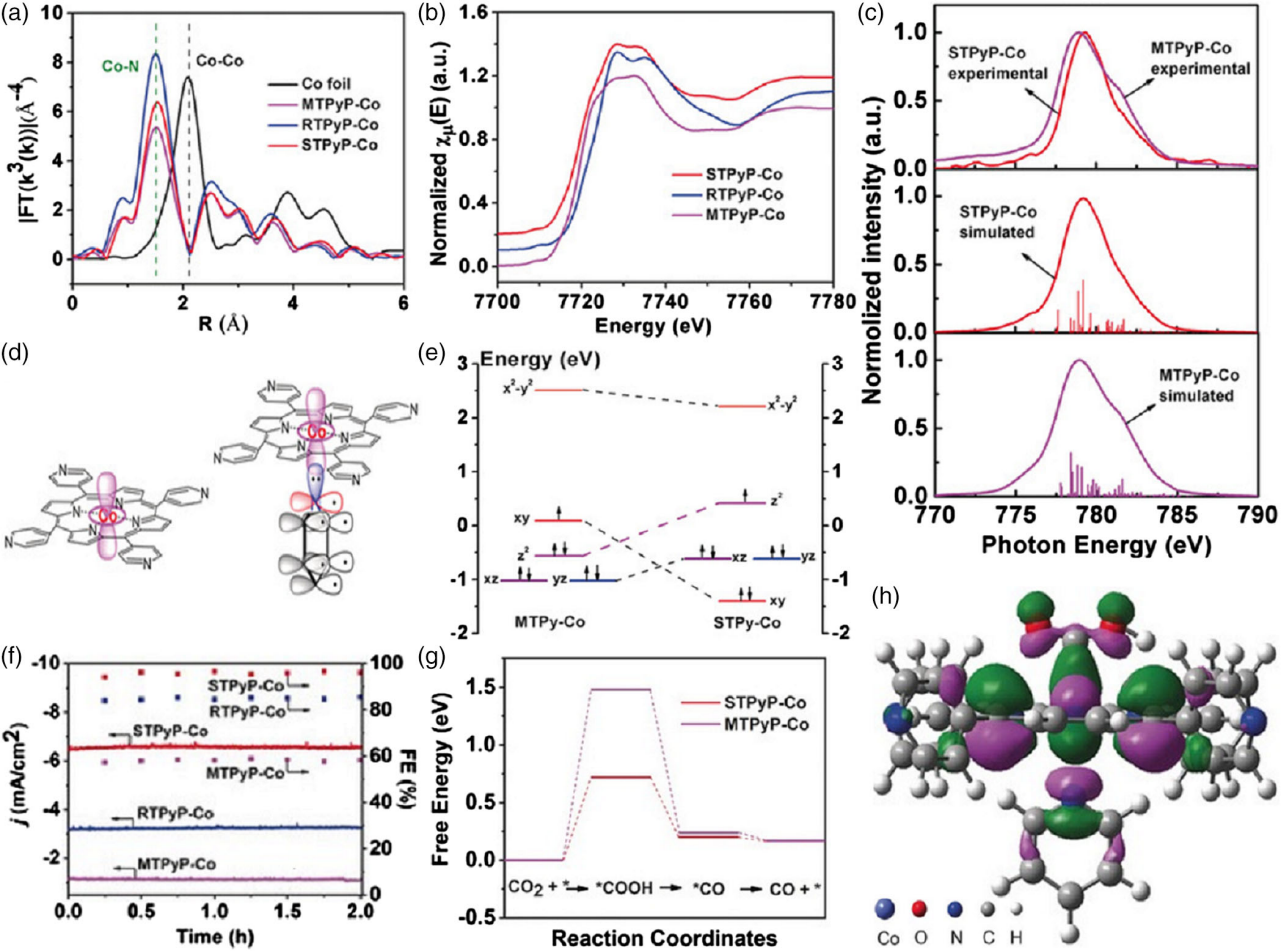
a) Ex situ EXAFS in *R*‐space of the four studied catalysts. b) The corresponding ex situ Co K‐edge XANES. c) Experimental and simulated Co L‐edge absorption of STPyP‐Co and MTPyP‐Co. d) Interaction between pyridine and central Co. e) Co 3d orbital splitting of Co centers in MTPyP‐Co and STPyP‐Co obtained from multiple fitting of Co L‐edge absorption. f) Current density and FE_CO_ of STPyP‐Co at −0.62 V. g) Calculated free‐energy states of the CO_2_ reduction to CO on STPyP‐Co. h) Spatial representation of highest occupied molecular orbital (HOMO) of [STPyP‐Co‐COOH] intermediates. a–h) Reproduced with permission.^[19g]^ Copyright 2019, Wiley‐VCH.

As a typical product of CO_2_ conversion, CO can be directly used to create multiple complex carbon‐based fuels and feedstocks, such as the methanol generated by hydrogenation through gas‐to‐liquid conversion reactions and the liquid hydrocarbon fuels produced via the Fischer–Tropsch process.^[^ ^52^ ^]^ To this end, Han et al. successfully synthesized a free‐standing and ultrathin 2D MOF, tetra(4‐pyridyl) porphyrin cobalt(II) nanosheets (denoted as STPyP‐Co), adopting an axial coordination assembly approach for electrocatalytic CO_2_ reduction in a 0.5 m KHCO_3_ solution (**Figure** **10**).^[19g]^ Because of the unique energy level of metal d_z_2 orbital induced by its ultrathin structure, the as‐prepared MOF‐based catalyst exhibited greatly boosted electrocatalytic properties, including high activity, selectivity, and stability, for the conversion of CO_2_ to CO with an FE_CO_ of 96 % at an overpotential of 500 mV and a turnover frequency (TOF) of 4.21 s^−1^ over 48 h, which rivaled the state‐of‐the‐art catalysts.^[^ ^53^ ^]^ By combining XAS results and theoretical computation, the structure–activity relationship was unambiguously revealed. The superior CO_2_RR activity of STPyP‐Co was attributed to the decreased activation energy of RDS. Furthermore, the molecular orbital analysis suggested that the Co with raised d_z_2 orbital could be reduced more easily by filling d_z_2, leading to an excellent electrocatalytic CO_2_RR performance. This work not only offered a general bottom‐up synthetic strategy for creating heterogeneous pristine MOF‐based catalysts with the optimized catalytic CO_2_RR activities at the molecular orbital level but also laid the foundation toward the establishment of the structure–activity relationship.

To achieve an effective electroreduction of CO_2_ into CO using pristine MOFs, introducing external fields such as light irradiation also offers an innovative platform toward optimizing catalytic pathways and properties by altering electrocatalysts’ electronic performances, including electron transfer, band bending, charge distribution, Fermi level, and desorption energy of intermediates.^[^ ^54^ ^]^ Based on this, Wang and co‐workers recently mimicked the structure of chlorophyll to construct a series of zirconium porphyrinic MOF hollow nanotubes as photo‐coupled electrocatalysts for CO_2_RR.^[^ ^55^ ^]^ Under visible light input, the as‐synthesized pristine MOF‐based materials presented a high FE_CO_ of 95.2% and an ultrahigh TOF of 37 069 h^−1^ with a much positive shift of overpotential (130 mV), nevertheless, the similar value of TOF could only be achieved at −1.1 V in the absence of light irradiation, where coordinated metal atoms served as active centers and porphyrin ligand acted as a light switch to collect photons and stimulate external electron transfer from the ground state to T1 state. With the help of experimental results and DFT computational analysis, this work successfully verified the high feasibility of “photo‐coupled pristine MOF electrocatalysis” and provided a unique route for CO_2_ electroreduction at a low overpotential.

**Figure 11:**
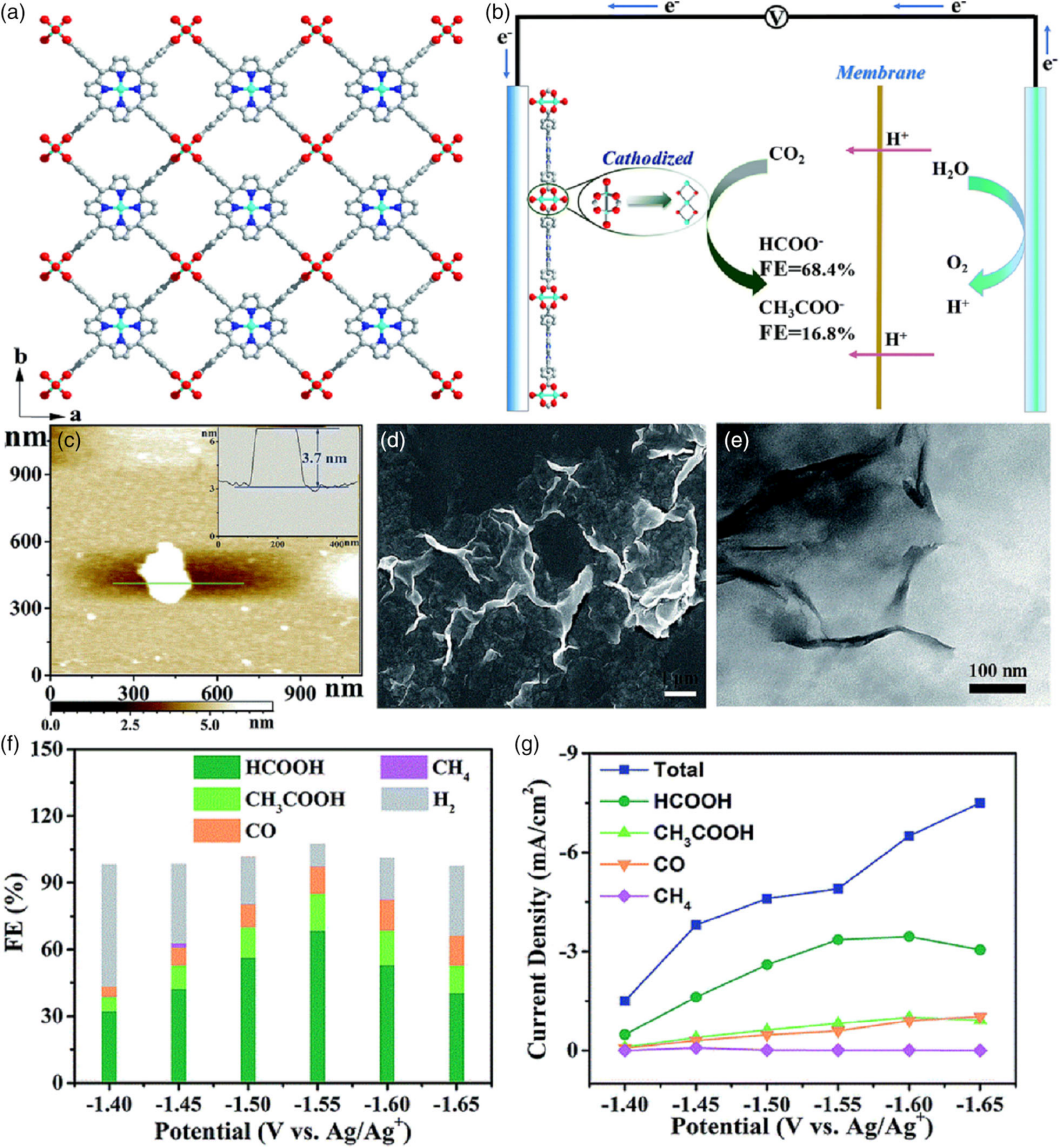
a) Crystal structure of Cu_2_(CuTCPP) nanosheets along the *c*‐axis (red: O; blue: N; gray: C; cyan: Cu). b) CO_2_RR system with Cu_2_(CuTCPP) nanosheets as the catalyst. c) Atomic force microscopy (AFM) image of the nanosheets with the thickness curve (inset). d) SEM image of Cu_2_(CuTCPP) nanosheets. e) TEM image of Cu_2_(CuTCPP) nanosheets. f) FE of the pre‐electrolyzed Cu_2_(CuTCPP) nanosheets in CO_2_‐saturated CH_3_CN electrolyte with 1 m H_2_O and 0.5 m EMIMBF_4_. g) Total and partial current densities for the CO_2_RR products on the pre‐electrolyzed Cu_2_(CuTCPP). a–g) Reproduced with permission.^[19h]^ Copyright 2019, The Royal Society of Chemistry.

In addition to CO products, a series of 2D Cu_2_(CuTCPP) MOFs, porphyrin MOF nanosheets based on copper(II) paddle wheel cluster, were recently constructed and then used for efficient electroreduction of CO_2_ to high‐value products, such as formate and acetate (**Figure** **11**).^[19h]^ In particular, the cathodized Cu_2_(CuTCPP) MOF‐based catalysts were able to display a significantly improved activity and selectivity for the electrochemical conversion of CO_2_ to formate with an FE of 68.4% at around −1.85 V. Furthermore, the *FE* for the electroreduction of CO_2_ into two liquid products (i.e., formate and acetate) reached 85.2% with a total current density of 4.5 mA cm^−2^. The combination of X‐ray diffraction (XRD), XPS, FTIR, and HRTEM studies demonstrate that the Cu(II) carboxylate nodes are likely to be transformed into the heterostructures of CuO, Cu_2_O, and Cu_4_O_3_ through the Cu(HCOO)_2_ and Cu(OH)_2_ intermediates, thereby significantly facilitating the CO_2_RR process due to the synergistic enhancement of porphyrin‐Cu(II) complexes. Significantly, this work proposed a new‐found concept “predesign” for the intelligible design of high‐performance pristine MOF‐based electrocatalysts.

## 4 MOF‐Based Host–Guest Composites for Electrocatalysis

To meet the stringent requirements in the practical application of MOFs, in addition to focusing on the regulation of pristine MOFs themselves, governable incorporation of various functional guest materials and pristine host MOFs, namely, enclosing catalytically active materials into/onto the pores, matrices, or layers of MOFs, is able to enrich the features of MOFs by taking their essence and discarding their dregs, leading to both their activity enhancement and framework stabilization.^[^ ^56^ ^]^ In 2014, Allendorf's group reported a “star” Cu‐based MOF, HKUST‐1 doped with an organic semiconductor 7,7,8,8‐tetracyanoquinodimethane (TCNQ), which was the first MOF possessing tunable electrical conductivity in line with the “Guest@MOF” concept.^[^ ^57^ ^]^ This work certainly laid a solid foundation toward the following‐up development of MOF‐based host–guest composite catalysts (MOF‐HGCCs) for accelerating various key reactions (involving but not limited to the fuel cell, water splitting, and CO_2_ reduction) of renewable energy conversion and storage. The well‐defined porous structures of pristine MOFs render themselves superb candidates for hosting/supporting different guest species, such as metal, metal oxides/hydroxides, quantum dots, carbon materials, polymers, diverse functional molecules, and so on. Therefore, the resultant MOF‐HGCCs with intrinsic pristine MOFs’ merits and implemented additional properties provide a brand‐new avenue to realize the significantly boosted catalytic activities, selectivity, and stability for highly efficient MOF electrocatalysis, where the pristine MOFs and introduced/foreign materials are hosts and guests, respectively.^[^ ^58^ ^]^ By dividing MOF‐HGCCs into two groups according to whether they contain precious metals or not, their recent advancements related to the design, structure–performance relationship exploration, and practical application are systematically and clearly summarized in the following two subsections.

### 4.1 Precious‐Metal‐Based MOF‐HGCCs

To surmount both some inherent limitations of pristine MOFs (such as low electrical conductivity and the blockage of catalytically active sites by organic linkers) and the major challenge of scale‐up of high‐performance precious‐metal‐based electrocatalysts caused by the high price and scarcity of precious metals, ones managed to adopt pristine MOFs as host materials to support and stabilize guest precious metal (e.g., Pt, Ru, Pd, Au, Ir, etc.) species. Hence, a variety of precious‐metal‐containing MOF‐based composites/hybrids have been created as highly active and stable electrocatalysts. Taking advantage of the ultrahigh activity of abundant foreign precious‐metal‐based active centers and substantially reduced utilization of PGM, precious‐metal‐based MOF‐HGCCs, thus, hold great promise to replace the expensive commercial precious‐metal‐based catalysts, such as Ir/C and Pt/C catalysts. Hence, it is urgently needed to develop various high‐performance MOF‐HGCCs with ultralow content of precious metals for the practical application involved in diverse key reactions (such as ORR, HOR, HER, OER, CO_2_RR, and so on) of sustainable energy conversion and storage.

**Figure 12:**
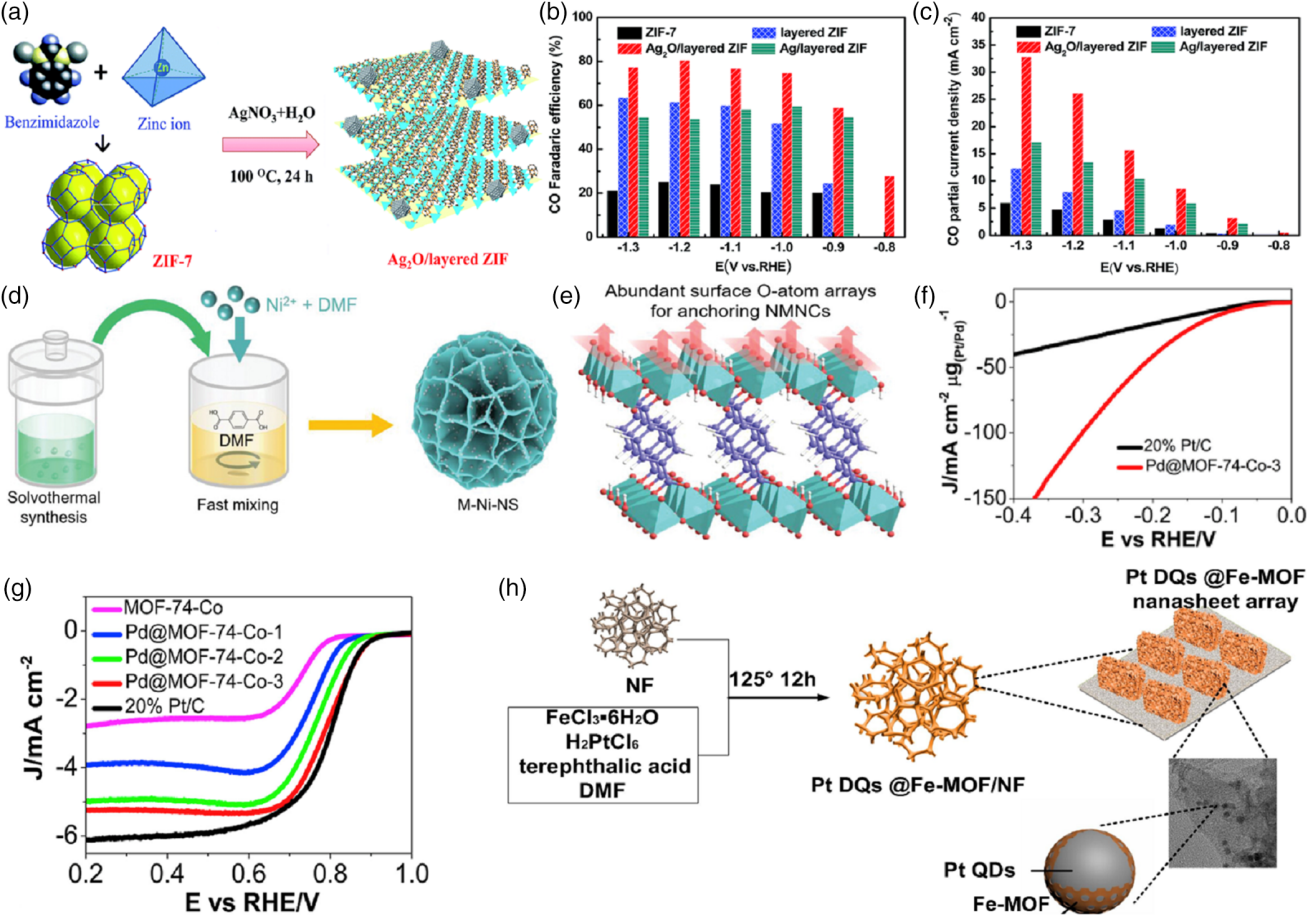
a) Schematic illustration of Ag_2_O@layered ZIF synthesis. b) FEs of different catalysts for CO production. c) The corresponding CO_2_RR partial current densities. a–c) Reproduced with permission.^[^ ^59^ ^]^ Copyright 2017, The Royal Society of Chemistry. d) Schematic synthesis of M—Ni—NS. e) Structural model showing abundant O‐atom arrays on Ni—NS surface for anchoring NMNCs. d,e) Reproduced with permission.^[^ ^61^ ^]^ Copyright 2021, Wiley‐VCH. f) HER polarization curves of the Pd@MOF‐74 and Pt/C in a 0.5 m H_2_SO_4_ solution with a scan rate of 10 mV s^−1^. g) ORR polarization curves of different samples in a 0.1 m KOH solution with 10 mV s^−1^ at 1600 rpm. f,g) Reproduced with permission.^[^ ^63^ ^]^ Copyright 2019, Elsevier Inc. h) Illustration for the preparation of core–shell Pt DQs@Fe‐MOF. Reproduced with permission.^[^ ^64^ ^]^ Copyright 2019, Elsevier Inc.

To optimize the electrocatalysis efficiency of this type of catalytic materials for critical reactions, enormous efforts have been devoted to tuning their sizes, morphologies, and compositions. As an example of monofunctional MOF‐HGCCs containing precious metals for boosting electrochemical CO_2_ conversion, the guest Ag_2_O nanoparticles were immobilized into the host‐layered ZIF‐7 to synthesize Ag_2_O@ZIF hybrids via a simple one‐pot hydrothermal treatment (**Figure** **12**a).^[^ ^59^ ^]^ As presented in Figure 12b,c, the resulting Ag‐based MOF‐HGCCs showed an FE_CO_ of 80.5% with a current density of 26.2 mA cm^−2^ at −1.2 V, apparently outperforming pure‐layered ZIF‐7 (25.0% and 4.7 mA cm^−2^) and commercial Ag/C catalyst (36.4% and 6.4 mA cm^−2^) for CO_2_RR. This can be explained by the synergistic effects between Ag_2_O nanoparticles and layered ZIFs, as well as the facilitated mass transport in consequence of the well‐modified specific surface area of layered Ag_2_O@ZIF. This work offered an alternative route toward designing MOF‐based catalysts for CO_2_RR using pristine MOFs as substrates to anchor silver active species. From the perspective of both the morphology and size control, a spherical palladium‐based MOF (denoted as Pd@MOF) with a small radius of 2.2 μm and a low Pd loading was recently synthesized via a facile two‐step approach based on the host–guest chemistry.^[^ ^60^ ^]^ The as‐prepared MOF‐HGCC achieved remarkable promotion for H_2_ generation via a Volmer–Heyrovsky mechanism with an exceptional electrocatalytic HER performance under an acidic environment, which was ascribed to the intrinsically high porosity and large specific surface area of MOF as well as the synergistic effect between Pd and MOF. On the other hand, the distribution and catalytic activities of noble metal components also play a crucial role in the enhancement of precious‐metal‐based MOF‐HGCCs’ cost‐effectiveness. More recently, Jiang et al. reported a series of hybrids of noble metal nanocrystals (NMNCs) and ultrathin 2D MOF nanosheets with admirable dispersity and stability (Figure 12d).^[^ ^61^ ^]^ As suggested in Figure 12e, they were constructed by a multiscale optimization strategy with the help of abundant O‐atom arrays on the 2D MOF nanosheets surface for efficient OER in basic media, denoted as M—Ni—NS (M = Ir, Ru, or Pt). Among them, Ir‐based MOF‐HGCCs displayed a boosted activity with a low overpotential of 270 mV at 10 mA cm^−2^ and long‐term stability. Through the combination of experimental results and computational study, it was unveiled that the M—O—Ni bridging bonds, originated from the significant metal–support interaction (MSI) from the intimate contact between parent materials, were mainly responsible for the improvement of catalytic performances by tailoring their active sites’ electronic structure and enhancing their conductivity.

In addition to functioning in a single reaction, MOF‐HGCCs with various noble metals are likely to act as efficient bifunctional electrocatalysts for ORR/HER, water splitting (HER/OER). For example, Ganesan and co‐workers recently demonstrated a simple synthesis approach for fabricating Au@Zn‐MOF catalysts by embedding gold nanoparticles into a Zn‐based MOF.^[^ ^62^ ^]^ The Au‐based MOF‐HGCCs showed high catalytic activities for both ORR and HER in acidic electrolytes, in which ORR took place through a two‐electron ORR pathway with hydrogen peroxide (H_2_O_2_) as the final product, whereas HER proceeded via Volmer mechanism with adsorption of H^+^ on the active sites. More recently, Chen and co‐workers successfully enclosed foreign tiny Pd clusters in the cavities/pores of electrically conductive M‐MOF‐74 (M = Co, Ni, and Zn) to create Pd@MOF‐74‐M hybrids with uniformly distributed Pd metals and greatly lowered Pd loading, and then probed their catalytic properties toward HER and ORR.^[^ ^63^ ^]^ For HER, Pd@MOF‐74‐Co realized a comparable *E*_onset_ of −40 mV to the commercial 20% Pt/C catalyst (−32 mV) and distinctly higher current densities. For ORR, 8‐fold and 9.4‐fold higher mass activities than Pt/C were achieved at 0.8 and 0.85 V, respectively. Meanwhile, they also showed stronger robustness under both acidic and alkaline conditions. Thus, the Pd@MOF‐74‐Co hybrids were directly used as bifunctional electrocatalysts with superior electrocatalytic performances compared with commercial Pt/C catalysts for both HER and ORR because of strong host–guest interaction (Figure 12f,g). In another study, Ye et al. synthesized a high‐performance bifunctional MOF‐HGCC using a generic one‐step hydrothermal treatment for water splitting, in which Pt quantum dots cores were homogeneously integrated with Fe‐MOF nanosheet arrays shell (Pt QDs@Fe‐MOF) with a porous cuboids structure on Ni foam (Figure 12h).^[^ ^64^ ^]^ Benefiting from the quantum size and core–shell structure, whereas the Pt QDs@Fe‐MOF electrocatalyst possessed ultralow content of Pt (1.84 μg cm^−2^), the overpotentials of 33 and 191 mV were realized at 10 and 100 mA cm^−2^ in 1 m KOH solutions, respectively. Furthermore, the Pt‐based MOF‐HGCC electrodes showed exceptional activity and stability with a current density of 10 mA cm^−2^ at 1.47 V for overall water splitting of at least 100 h. This work proposed a unique porous core–shell structure to judiciously engineer the MOF‐HGCCs for water splitting in industrial practices.

### 4.2 Precious‐Metal‐Free MOF‐HGCCs

In recent years, researchers have been persistently committed to further improving the overall catalytic performances of MOF‐HGCCs and minimizing their costs as much as possible. Apart from the above‐mentioned good employment of precious‐metal‐based materials, the incorporation of foreign materials without precious metals into MOFs also provides an alternative direction for the synthesis of MOF‐HGCCs and simultaneously lowers the activation energy of catalytic reactions through host–guest cooperation within MOFs.^[^ ^65^ ^]^ Hence, the optimized precious‐metal‐free MOF‐HGCCs can be practically applied to diverse clean energy devices, such as fuel cells, electrolyzers, and metal–air batteries.

**Figure 13:**
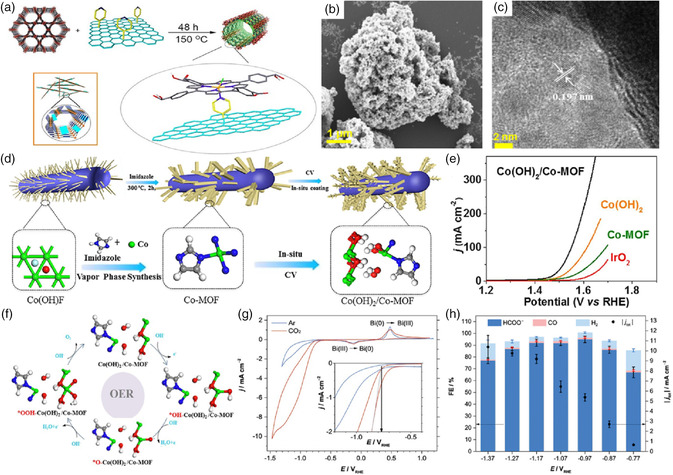
a) Schematic illustration of the preparation of PCN‐222‐G‐py. Reproduced with permission.^[^ ^68^ ^]^ Copyright 2016, Wiley‐VCH. b) SEM image of 40 wt% MoS_*x*_/Ni‐MOF‐74 composite. c) HRTEM image of 40 wt% MoS_*x*_/Ni‐MOF‐74 composite. b,c) Reproduced with permission.^[^ ^70^ ^]^ Copyright 2021, John Wiley & Sons. d) Schematic illustration of the synthesis of Co(OH)_2_/Co‐MOF. e) linear sweep voltammetry (LSV) curves of different catalysts with a scan rate of 1 mV s^−1^. f) Free energy diagram of OER on Co‐MOF, Co(OH)_2_, and Co(OH)_2_/Co‐MOF. d–f) Reproduced with permission.^[^ ^72^ ^]^ Copyright 2021, Elsevier Inc. g) Cyclic curves of Bi(btb) at 50 mV s^−1^ in Ar‐ and CO_2_‐saturated 0.5 m KHCO_3_ solutions (inset: how the value of *E*_onset_ was determined). h) CO_2_RR results at various potentials in CO_2_‐saturated electrolytes based on 30 min tests. g,h) Reproduced with permission.^[^ ^76^ ^]^ Copyright 2020, Wiley‐VCH.

In 2015, Li and co‐workers creatively stabilized ϵ‐MnO_2_ nanorods on an Fe‐MOF support to prepare a highly porous ϵ‐MnO_2_/MOF(Fe) composite for oxygen reduction in alkaline solutions.^[^ ^66^ ^]^ The resulting Mn‐based MOF‐HGCCs yielded a superior activity and stability than those of ϵ‐MnO_2_ during the ORR process, even comparable to the commercial 20% Pt/C, in which ORR favored an obvious four‐electron transfer pathway. To further enhance the ORR activity of MOF‐HGCCs, various amounts of CuS nanoparticles were inserted in 3D Cu‐MOFs [Cu_3_(BTC)_2_⋅(H_2_O)_3_] (BTC = 1,3,5‐benzenetricarboxylate) to construct CuS@Cu‐BTC composites with exponentially increased (109‐fold) conductivity.^[^ ^67^ ^]^ In spite of the poor porosity obtained, the resultant material exhibited a boosted ORR activity by a quasi‐four‐electron pathway in terms of its *E*_onset_ of 0.91 V and a kinetic current density of 11.3 mA cm^−2^ at 0.55 V. Except for the introduction of metal‐based materials into MOFs for alkaline ORR, Sohrabi et al. recently proposed a bioinspired heme‐like MOF‐HGCC through an amazing assembly of pyridine‐functionalized graphene (G‐py) and a 3D MOF for promoting acidic ORR (**Figure** **13**a).^[^ ^68^ ^]^ Because of the favorable role of a stable porous coordination network (PCN‐222) in tuning electronic and geometric structures of active metal centers, the ORR process was markedly accelerated with excellent stability in acidic media. These works opened the door for the rational design of inexpensive and highly stable next‐generation ORR electrocatalysts for fuel cells without precious metals and the pyrolysis process.

To achieve highly efficient water splitting, various MOF‐HGCCs without precious metals have been developed to facilitate half‐reactions, i.e., hydrogen evolution and/or oxygen evolution. As a promising example for HER, a copper(II)phthalocyanine‐incorporated MOF‐HGCC (denoted as CuPc@MOF) was recently synthesized with an exceedingly boosted HER activity compared with the corresponding pristine MOF.^[^ ^69^ ^]^ Also, the synergetic effect of the CuPc crystals on the MOF matrix was revealed as the main justification for its performance improvement. Most recently, Do et al. followed host–guest chemistry using a simplistic solvothermal strategy to develop cost‐effective HER catalysts by combining amorphous molybdenum sulfide (MoS_*x*_) and Ni‐MOF‐74 (Figure 13b,c).^[^ ^70^ ^]^ They also suggested that NiMoS phases can lower the hydrogen adsorption energy on the catalysts, leading to an excellent hydrogen generation performance of the optimal MoS_*x*_/Ni‐MOF‐74 in acidic media (40 wt% MoS_*x*_, *E*_onset_ = −114 mV and Tafel slope = 53.1 mV dec^−1^). Toward the OER process, Zhou and co‐workers recently constructed a series of single‐atom implanted 2D MOF‐HGCCs (labeled as Ni@HUST‐8) as efficient electrocatalysts for water oxidation in basic media, which exhibited better catalytic properties than commercial IrO_2_.^[^ ^71^ ^]^ In 2021, Yao et al. reported an in situ cathodic electrotransformation method to synthesize a cobalt hydroxide‐coated Co‐MOF (named Co(OH)_2_/Co‐MOF) as the latest precious‐metal‐free MOF‐HGCCs for OER electrocatalysis (Figure 13d–f).^[^ ^72^ ^]^ Experimental analysis and DFT calculations revealed that the enhanced OER performance of the as‐prepared material including long‐term stability and high activity with an overpotential of 196 mV at 10 mA cm^−2^ for 15 h continuous testing in a 1 m KOH solution was ascribed to the tailored adsorption free energy of oxygenic intermediates and unique structure with abundant exposed active centers and well‐tuned gas release ability. This work laid the foundation for the future design of MOFs with coordinatively saturated metal centers for OER. In another study, the boosted overall water splitting including HER and OER can be attained by a low‐cost, highly active, and stable Co‐based MOF‐HGCC (Co_3_O_4_@Co‐MOF).^[^ ^73^ ^]^ The dual‐function MOF‐based hybrid was easily produced by confining guest Co_3_O_4_ nanocubes on the surface of the Co‐MOF nanosheet in the light of a facile one‐pot hydrothermal method, which had great potential to be extended to the fabrication of other metal oxides/hydroxides@MOF composite materials.

As one of the high‐efficiency heterogeneous electrocatalysts with minimal cost, the precious‐metal‐free MOF‐HGCC has also turned out to be an encouraging candidate for electroreduction of CO_2_. For instance, Kung et al., for the first time, immobilized copper(II) clusters into the solvothermally grown thin film of a zirconium MOF (NU‐1000) by a solvothermal deposition method to synthesize a single‐site Cu‐based MOF‐HGCC for CO_2_ reduction in 2017.^[^ ^74^ ^]^ In particular, the metallic Cu nanoparticles enshrouded within the hexagonal channels of NU‐1000 were electrochemically addressable and demonstrated an impressive electrocatalytic performance toward CO_2_ conversion with formate as the major product, in which the crystallinity and morphology of this MOF‐HGCC remained unchanged after the electrocatalysis. Furthermore, they found that the size of the MOF channels exerted decisive impacts on the size of copper nanoparticles acquired using this method. More recently, Qiu and co‐workers reported another Cu‐based MOF‐HGCC toward efficient CO_2_ conversion by a time‐resolved controllable restructuration from Cu_2_O to Cu_2_O@Cu‐MOF in the presence of the 1,3,5‐tricarboxylic acid (H_3_BTC) ligand.^[^ ^75^ ^]^ Armed with ultrahigh specific surface area for enhanced CO_2_ adsorption capacity and the boosted charge transfer bought by sufficient Cu_2_O active sites, the as‐prepared MOF‐based hybrid possessed superior electrocatalytic CO_2_RR selectivity with a high FE of 79.4% for hydrocarbon (CH_4_ and C_2_H_4_) at −1.71 V, especially 63.2% FE for CH_4_. Besides embedding copper‐based materials to modify MOFs for electroreduction of CO_2_, a Bi‐based MOF‐HGCC (Bi(btb)) was recently constructed by the incorporation of Bi^3+^ cations into a porous MOF scaffold.^[^ ^76^ ^]^ Encouragingly, it delivered a high selectivity for conversion of CO_2_ to formate with an FE as large as 95% at an overpotential of 770 mV (Figure 13g,h). Experimental characterization results revealed that the periodic arrangement of Bi cations in highly porous Bi(btb) determined the well‐dispersed Bi nanoparticles, which were exposed as active sites for CO_2_RR electrocatalysis. Inspired by these works, whereas pristine MOFs’ large‐scale use was usually restricted by their inherently low electronic conductivity, applying them as a robust platform to confine active sites into organic mesh certainly offers a new route of developing low‐cost, high‐performance, and earth‐abundant MOF‐HGCCs for electrocatalysis.

## 5 Conclusions and Perspectives

As an emerging class of electrocatalysts, MOFs have great potential to replace or complement current noble metal‐based and carbon‐based catalysts due to their ultrahigh specific surface area, interconnected porosity, and accessible metal sites, as well as high controllability in both components and structures. The development of highly efficient and inexpensive pristine MOFs and MOF‐HGCCs for electrocatalysis involved in the key reactions (i.e., ORR, HOR, HER, OER, and CO_2_RR) of renewable energy conversion and storage has become an emerging research field in the past few years. In this article, we summarize the unique merits of MOF‐based electrocatalysts associated with their material design principles and mechanism discussion. Also, a comprehensive but critical review of recent advances in MOF electrocatalysis, including oxygen reduction, hydrogen oxidation, water splitting, and CO_2_ reduction, is provided.

Although tremendous progress has been made, the study on MOF electrocatalysis is still in its infancy, and many challenges (such as low electrical conductivity, bad contact efficiency, and poor stability) are becoming the major limitations for the performance enhancement of MOF‐based materials and awaiting to be overcome urgently. Herein, a comparison of the best catalytic performances of pristine MOFs and other materials is presented in **Table** **2**. Encouragingly, it can be seen clearly from this comprehensive comparison that the electrocatalytic performances of many pristine MOFs in different reactions (e.g., ORR, HER, OER, and CO_2_RR) are comparable to, or even superior to the best catalytic performances of other types of state‐of‐the‐art materials (e.g., metal oxides, metal hydroxides, metal sulfides, commercial PGM, perovskites, carbon, etc.). However, some of the pristine MOFs’ activities and selectivity are still not as decent as other kinds of materials in terms of their specific *E*_onset_, *E*_1/2_, *η*_10_, FE, and so on, suggesting that they have not completely overtaken other latest high‐performance catalytic materials. Several factors restricting MOFs’ performances and large‐scale practical application are emphasized here as follows. First, in terms of material design, current research about the MOF synthesis mainly focuses on size control. The works related to dimension control are relatively rare. Nevertheless, in general, the ultrathin nanosheets and nanowires could exhibit a better catalytic performance owing to the enhanced conductivity, optimized contact efficiency, and increasing exposed active sites. Second, the stability of MOFs is another big concern in the practical application of electrocatalysis, because many relevant and important reactions occur in harsh electrolytes such as strong alkaline/acidic media. Third, the MOFs with uniform distribution of metal atoms theoretically might realize the maximum atom utilization. However, the metal sites in many MOFs are blocked by the ligands/solvents, leading to their much‐decreased accessibility and poor contact efficiency. Finally, despite numerous studies for developing high‐performance MOF‐based electrocatalysts, these fundamental questions such as the electron transfer process through the MOF scaffolds and the specific role of organic ligands are still unclear. In conclusion, this situation needs to be further improved by rationally designing and judiciously engineering the morphologies, sizes, structures, and components of MOF‐based materials to realize the targeted electrocatalytic functions.

**Table 2:** Comparison of the best catalytic performances of pristine MOFs and other types of state‐of‐the‐art electrocatalysts for various key reactions

Electrocatalystsa)	Reactions	Electrolytes	*E*_onset_ [V]	*E*_1/2_ [V]	*J*_L_ b) [mA cm^−2^]	*η*_10_ c) [mV]	FE [%]	*k* d) [mV dec^−1^]	Ref.
Ni_3_(HITP)_2_	ORR	0.1 m KOH	0.82	–	–	–	–	128	[30]
PcCu—O_8_—Co	ORR	0.1 m KOH	–	0.83	5.30	–	–	61	[31]
I_2_&ZIF‐67	ORR	0.1 m KOH	0.90	0.83	–	–	–	38	[32]
Fe—N—C	ORR	0.1 m KOH	0.90	0.80	5.60	–	–	–	[77]
FeN_3_P‐SAC	ORR	0.1 m KOH	0.94	0.87	5.66	–	–	–	[78]
Fe/N‐CNFs	ORR	0.1 m KOH	0.88	0.79	–	–	–	–	[79]
Co_4_N/C	ORR	0.1 m KOH	–	0.88	5.50	–	–	49	[80]
LaMnO_3_	ORR	0.1 m KOH	0.83	0.63	5.9	–	–	–	[81]
PdCu/C	ORR	0.1 m KOH	–	0.89	–	–	–	36	[82]
MnV/N,S‐CNTs	ORR	0.1 m KOH	0.95	0.84	5.17	–	–	92	[83]
20 wt% Pt/C	ORR	0.1 m KOH	0.94	0.83	4.86	–	–	91	[84]
Fe_3_C/C‐700	ORR	0.1 m HClO_4_	0.90	0.73	–	–	–	–	[85]
Ti_0.8_Co_0.2_N	ORR	0.1 m HClO_4_	0.96	0.79	5.65	–	–	–	[86]
NiCo‐N‐CNFs	ORR	0.1 m KOH	0.91	0.82	5.01	–	–	72	[84]
	OER	0.1 m KOH	–	–	–	161	–	89	
NiFe‐NDC/NF	ORR	0.1 m KOH	0.92	0.83	–	–	96	70	[13c]
	OER	0.1 m KOH	–	–	–	–	99.5	68	
NiCo‐UMOFN	OER	1 m KOH	1.39	–	–	189	–	42	[7]
Ni_0.9_Fe_0.1_‐MOF	OER	1 m KOH	1.35	–	–	198	≈100	–	[47]
NiFeCo‐MOF/NF	OER	1 m KOH	–	–	–	257	–	41	[48]
RuO_2_	OER	0.1 m KOH	–	–	–	162	–	–	[84]
V‐doped FeOOH	OER	1 m KOH	1.48	–	–	390	–	37	[87]
FeNi LDH/G	OER	1 m KOH	1.43	–	–	195	–	35	[88]
NiCo‐LDH	OER	1 m KOH	–	–	–	367	–	40	[89]
Ni‐PNPs	OER	1 m KOH	1.48	–	–	300	–	64	[90]
Pt/NiO	OER	1 m KOH	–	–	–	358	–	33	[91]
Fe‐N_4_ SAs/NPC	OER	1 m KOH	–	–	–	430	–	95	[92]
NENU‐500	HER	0.5 m H_2_SO_4_	0.18	–	–	237	–	96	[14c]
AB/CTGU‐5	HER	0.5 m H_2_SO_4_	0.02	–	–	44	–	45	[16e]
1T‐MoSe_2_ NS	HER	0.5 m H_2_SO_4_	–	–	–	152	–	52	[93]
NiSe_2_/Ti	HER	1 m KOH	–	–	–	96	–	82	[94]
CoSe_2_ NW/CC	HER	0.5 m H_2_SO_4_	–	–	–	130	–	32	[95]
MoS_2_/GF	HER	0.5 m H_2_SO_4_	0.07	–	–	100	–	41	[96]
Co PCN	HER	1 m KOH	0.04	–	–	89	–	52	[97]
Pt/MoS_2_	HER	0.1 m H_2_SO_4_	–	–	–	60	–	96	[98]
Co@BCN	HER	0.5 m H_2_SO_4_	–	–	–	96	–	64	[99]
	OER	1 m KOH	–	–	–	183	–	73	
Ni(II)‐MOF	HER	1 m KOH	–	–	–	101	–	51	[14d]
	OER	1 m KOH	–	–	–	219	–	48	
STPyP‐Co	CO_2_RR	0.5 m KHCO_3_	–	–	–	–	96%, CO	–	[53a]
Cu_2_(CuTCPP)	CO_2_RR	CH_3_CN/0.5 m EMIMBF_4_	–	–	–	–	68.4%, formate	–	[19h]
Ni—N—C	CO_2_RR	0.5 m KHCO_3_	–	–	–	–	92%, CO	–	[100]
Au NWs	CO_2_RR	0.5 m KHCO_3_	–	–	–	–	94%, CO	–	[101]
NSHCF	CO_2_RR	0.1 m KHCO_3_	–	–	–	–	94%, CO	–	[102]
In_2_O_3_‐rGO	CO_2_RR	0.1 m KHCO_3_	–	–	–	–	84.6%, HCOOH	–	[103]
Cu_0.8_In_0.2_	CO_2_RR	0.5 m NaHCO_3_	–	–	–	–	90%, CO	–	[104]
Cu_2_O/CuO@Ni	CO_2_RR	0.5 m KHCO_3_	–	–	–	–	95%, CO	–	[105]

a) SAC = single‐atom catalyst; CNFs = carbon nanofiber aerogels; CNTs = carbon nanotubes; NF = Ni foam; LDH = layered double hydroxide; G = graphene; PNPs = phosphides nanoplates; SAs = single atoms; NPC = *N*‐doped porous carbon; NS = nanosheet; NW = nanowires; CC = carbon cloth; GF = graphene film; PCN = phosphorized carbon nitride; BCN = N, B co‐doped ultrathin carbon cages; NSHCF = N and S co‐doped, hierarchically porous carbon nanofiber; rGO = reduced graphene oxide;

b) *J*_L_ for limiting current density;

c) *η*_10_ for overpotential required for the current density of 10 mA cm^−2^;

d) *k* for Tafel slope.

To better solve the aforementioned problems, the following suggestions or research directions are outlined. First, it is imperative to develop effective strategies to achieve precise control over MOFs in terms of components, morphologies, sizes, and thicknesses. Compared with the bulk 3D structures or micrometer‐sized particles, the low‐dimensional MOFs including ultrathin nanosheets or nanowires have a unique advantage in contact efficiency owing to their good film‐forming characteristics and anisotropy. Also, modifications in length and surface group of ligands are suggested to regulate the stability of the MOFs during the catalytic process. For example, MOFs coordinated with shorter ligands such as terephthalic acid usually provide good stability in alkaline solutions, whereas longer ligands (e.g., 4,4′‐diiodo‐2,2′,5,5′‐tetramethyl‐1,1′‐biphenyl and hexaaminobenzene) will greatly improve their acid resistance.^[24a]^ More importantly, the related formation mechanism of MOFs needs to be elucidated, which will lay a foundation for the functionalization of MOFs. Second, defect or coordination unsaturation engineering is expected to act as an effective approach to activate the metal sites inside MOFs. Defect engineering could not only further improve the catalytic activities of MOFs but also endow them with new functions. Third, the method of integrating pristine MOFs and foreign catalytic materials to construct MOF‐based composites is also an effective strategy to greatly improve the conductivity of as‐fabricated MOF‐based materials and produce strong interactions between MOFs and the introduced materials, further optimizing the electrocatalysis efficiency based on their enhanced activities and stability. Fourth, pristine MOFs have been used as outstanding electrocatalysts for ORR, HOR, water splitting, and CO_2_RR. However, so far, there is no reported work on the use of MOFs in other important industrial processes, such as chlorine evolution reaction (the core reaction of alkaline chlorine process) and nitrogen reduction reaction (NRR). It is necessary to extend the application of pristine MOFs. Fifth, to both clearly and comprehensively characterize MOF‐based catalysts, HRTEM and in situ spectroscopy techniques (such as XPS, FTIR, Raman, isotope tracing experiments, and XAS analysis involving extended X‐ray absorption fine structure [EXAFS] and X‐ray absorption near edge structure [XANES]) are frequently adopted for identifying the geometric and electronic structures, disclosing the catalytic mechanisms, exploring the synergistic effects, and further optimizing the catalyst structures. Especially, the XAS analysis has been extensively applied to determine the oxidation state of metal centers, bond length, short‐range disorder, coordination number, and local geometry. Sixth, the computational simulations are exceedingly useful to understand the mechanisms related to electrocatalytic activities and stability. Armed with such novel knowledge acquired from experiments and computational calculations, ones will be able to easily functionalize MOFs at the molecular/atomic level and implement the advanced catalytic reactions with high catalytic activities, excellent selectivity, and ultralong‐term stability.

In summary, further in‐depth research efforts are needed to surmount the challenges discussed earlier to obtain low‐cost, high‐performance pristine MOF‐based electrocatalysts, and even MOF‐based composites, ultimately achieving the benchmarking electrocatalytic performances in the practical application of renewable energy conversion and storage.

## Conflict of Interest

The authors declare no conflict of interest.
